# BACH2 links β1-adrenergic receptor/β-arrestin1 signaling to MIAT to inhibit cardiac fibroblast activation and cardiomyocyte apoptosis

**DOI:** 10.1038/s41420-026-02985-4

**Published:** 2026-02-28

**Authors:** Bruno Moukette, Jian-peng Teoh, Waleed J. Hashmi, Satoshi Kawaguchi, Tatsuya Aonuma, Hamedane Moustapha, Steven S. Welc, Simon J. Conway, Suthat Liangpunsakul, Lei Yang, Ankit A. Desai, Il-man Kim

**Affiliations:** 1 Department of Anatomy, Cell Biology, and Physiology, Indiana University School of Medicine, Indianapolis, IN USA; 2 Herman B Wells Center for Pediatric Research, Indiana University School of Medicine, Indianapolis, IN USA; 3 Division of Gastroenterology and Hepatology, Indiana University School of Medicine, Indianapolis, IN USA; 4 Krannert Cardiovascular Research Center, Indiana University School of Medicine, Indianapolis, IN USA; 5 Roudebush Veterans Administration Medical Center, Indianapolis, IN USA; 6 Present Address: Internal Medicine Research Unit, Pfizer Inc., Cambridge, MA USA; 7 Present Address: Shenzhen Institute of Synthetic Biology, Shenzhen Institutes of Advanced Technology, Chinese Academy of Sciences, Shenzhen, China; 8 Present Address: Department of Emergency Medicine, Asahikawa Medical University, Asahikawa, Hokkaido Japan; 9 Present Address: Department of Internal Medicine, Division of Cardiology and Nephrology, Asahikawa Medical University, Asahikawa, Hokkaido Japan

**Keywords:** Cell signalling, Cardiovascular biology, Mechanisms of disease, Long non-coding RNAs

## Abstract

The myocardial infarction-associated transcript (MIAT), a conserved long noncoding RNA, is upregulated in failing human and murine hearts. We previously demonstrated that systemic or cardiomyocyte (CM)-restricted ablation of MIAT in mice attenuated maladaptive cardiac remodeling following myocardial infarction by suppressing the expression of proapoptotic and profibrotic genes. Despite growing evidence from human and rodent studies implicating MIAT in heart failure, the upstream regulatory pathways controlling its expression remain poorly defined. We hypothesized that MIAT is regulated either by β-arrestin1-mediated β_1_-adrenergic receptor protective signaling or by the transcription factor BTB domain and CNC homolog 2 (BACH2), which is downregulated in failing human and murine hearts. In this study, we show that treatment with the β-blocker carvedilol downregulates cardiac MIAT via β_1_-adrenergic receptor/β-arrestin1 signaling and concurrently upregulates BACH2. Mechanistically, our co-immunoprecipitation and electrophoretic mobility shift assays reveal that BACH2 forms a nuclear complex with β-arrestin1 and binds to conserved elements within the MIAT promoter. Using primary adult human cardiac fibroblasts (CFs) as well as human and rodent CMs, we further show that BACH2 represses profibrotic and proapoptotic MIAT expression, thereby inhibiting CF activation and CM apoptosis. Together, these findings identify a novel regulatory axis involving β_1_-adrenergic receptor/β-arrestin1 signaling, BACH2, and MIAT, highlighting its critical role in maladaptive cardiac remodeling.

## Introduction

In adult hearts, the absence of cardiomyocyte (CM) renewal results in the replacement of damaged CMs with scar tissue, contributing to cardiac fibrosis [1, 2]. Prolonged cardiac fibrosis arises when quiescent fibroblasts differentiate into pathologically activated myofibroblasts in response to cardiac injury, ultimately driving progressive left ventricular remodeling and chronic heart failure [3]. Strategies that enhance CM survival and suppress fibroblast activation have been shown to improve cardiac function following injury or stress [1–3]. Therefore, a comprehensive understanding of cardiomyogenesis in injured adult hearts and the identification of key regulators responsible for CM loss and fibroblast activation are essential for developing therapies aimed at preserving myocardial integrity in myocardial infarction (MI) and chronic heart failure (HF).

The BTB domain and CNC homolog 2 (BACH2), a member of the BACH family of basic leucine zipper transcription factors, contains variants for genetic risk of type 1 diabetes [4, 5]. Notably, BACH2 expression was significantly reduced in human hypertrophic hearts and in mouse hearts following transverse aortic constriction (TAC) compared to controls [6]. The same study reported that systemic or CM-specific knockdown of *Bach2* exacerbated TAC-induced HF in mice [6]. Consistent with findings showing that the flavonoid myricetin conferred protective effects in other rodent models of HF [7], myricetin was shown to mitigate TAC-induced HF in part by upregulating BACH2 [6]. Additionally, BACH2 level was inversely associated with diabetes-induced myocardial injury in humans. CM-specific overexpression of *Bach2* attenuated diabetic cardiomyopathy in mice, whereas CM-specific *Bach2* conditional knockout (KO) in mice worsened diabetic cardiac dysfunction [8]. Although BACH2 was identified as an antihypertrophic and antidiabetic factor in CMs and murine hearts, its role in cardiac fibroblast (CF) activation and CM apoptosis under ischemic stress remains poorly understood.

Long noncoding RNAs (lncRNAs) have emerged as key regulators of HF pathogenesis [9, 10]. One important mechanism involves the interplay among lncRNAs, microRNAs (small noncoding RNAs; miRNAs or miRs), and messenger RNAs (mRNAs), whereby lncRNAs function as competing endogenous RNAs that sequester miRs, thereby derepressing their target mRNAs [11]. Several lncRNAs have also been implicated in the clinical context of HF [12, 13]. We previously identified the conserved lncRNA myocardial infarction-associated transcript (MIAT) as a negative regulator of miR-150-5p (miR-150) [14], which was activated by the β-adrenergic receptor (βAR) antagonist (β-blocker) carvedilol via β-arrestin1 (β-arr1)-mediated β_1_AR protective signaling [15]. Using systemic KO and transgenic mouse models as well as CM-restricted conditional KO mice, we demonstrated that systemic or CM-derived MIAT exacerbated maladaptive post-MI remodeling, in part by inducing proapoptotic and profibrotic gene programs including *p53*, *Bak1*, *Hoxa4*, *Col3a1*, *Col6a1*, *Postn*, and *Snail* [14, 16]. Notably, miR-150 overexpression ameliorated MIAT-driven maladaptive post-MI remodeling [14], directly establishing a functional in vivo MIAT/miR-150 axis during HF. These cumulative findings position MIAT as a significant upstream inhibitor of β_1_AR/β-arr1-responsive miR-150 in the heart.

Clinically, gain-of-function single-nucleotide polymorphisms in MIAT have been associated with an increased risk of MI [17, 18]. MIAT was also overexpressed in patients with Chagas cardiomyopathy [19] and was significantly associated with maladaptive cardiac remodeling in patients with type 2 diabetes [20]. Moreover, rodent studies revealed that MIAT was upregulated in mouse models of MI, angiotensin II (AngII)- and isoproterenol (ISO)-induced cardiac hypertrophy, and diabetic cardiomyopathy [21–25]. MIAT knockdown also improved cardiac dysfunction and adverse cardiac remodeling in post-MI [23] and in diabetic hearts [21] as well as reduced ischemia/reperfusion-induced myocardial infarct size and apoptosis [22]. MIAT loss also attenuated AngII- and TAC-induced HF, in part, by blunting a CM hypertrophic gene program and enhancing CM contractility [26]. We also reported that MIAT was upregulated in CMs and CFs isolated from MI hearts [14]. MIAT was upregulated in cultured rodent CMs subjected to AngII, ISO, high glucose (HG), or hypoxia/reoxygenation (H/R) [21–25]. MIAT knockdown was also shown to inhibit neonatal rat ventricular CM apoptosis induced by HG [21] and H/R-induced embryonic rat myoblast apoptosis [22]. Other work showed that MIAT repressed miR-150 expression, thereby acting as a positive regulator of CM hypertrophy [24, 25]. MIAT was also upregulated in mouse CFs subjected to AngII, and MIAT knockdown inhibited CF activation by reducing collagen production and CF proliferation [23]. Cumulatively, these findings underscore the clinical relevance and potential therapeutic implications of MIAT regulatory mechanisms in HF. However, little is known about mechanisms that regulate MIAT expression and function, specifically, in hearts, CFs, and CMs.

Although β-arrs participate in diverse cytoplasmic signaling networks [27], they also play critical roles in the nucleus [28, 29]. Among the two ubiquitous β-arr isoforms, β-arr1 is the predominant mediator of nuclear signaling, as it lacks a nuclear export signal present in β-arr2 [30]. In the present study, we report four key findings: (I) BACH2 interacts with β-arr1 in the nucleus and binds to the MIAT promoter; (II) Carvedilol, via β-arr1-mediated β_1_AR protective signaling, downregulates cardiac MIAT while concomitantly upregulating BACH2; (III) BACH2 is downregulated in patients with heart failure with reduced ejection fraction (HFrEF) and in post-MI mouse hearts, but its expression is restored in ischemic CFs and CMs following carvedilol treatment, inversely correlating with MIAT expression; and (IV) BACH2 suppresses the profibrotic and proapoptotic effects of MIAT, thereby inhibiting CF activation and CM apoptosis. Mechanistically, BACH2 serves as a critical transcriptional hub mediating β_1_AR/β-arr1-driven repression of MIAT. Collectively, these findings provide the first evidence that carvedilol/β_1_AR/β-arr1 signaling, in conjunction with the transcriptional repressor BACH2, constitutes a novel upstream regulatory pathway of maladaptive MIAT. Our results highlight the carvedilol/β_1_AR/β-arr1/BACH2/MIAT axis as a promising therapeutic target for organ fibrosis and ischemic HF.

## Results

### Carvedilol downregulates cardiac MIAT in a β1AR– and β-arr1–dependent manner

**Fig. 1:**
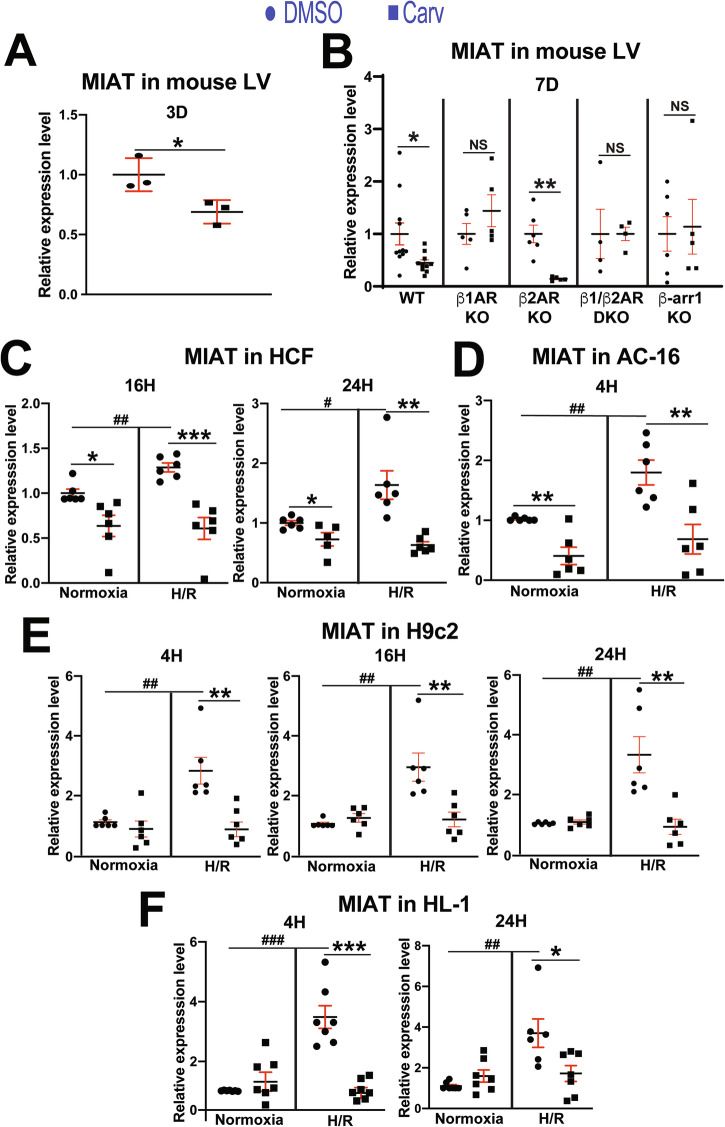
MIAT is downregulated in mouse left ventricles, human cardiac fibroblasts, and human and rodent cardiomyocytes by carvedilol. MIAT expression was detected by Real-time Quantitative Reverse Transcription (QRT)-PCR in left ventricles from adult mice stimulated with carvedilol (Carv: 19 mg/kg per day) or vehicle for 3 day (**A**) or 7 days (**B**). *N* = 3–11. Data are shown as fold induction of MIAT expression normalized to *Gapdh*. Unpaired 2-tailed *t*-test. *P < 0.05 or **P < 0.01 vs. DMSO. NS: not significant. **C**–**F** Primary adult human CFs (HCFs) as well as AC-16, H9c2, and HL-1 cardiomyocytes were treated with 1 μM Carv for 4–24 h (H) and subjected to either normoxia (basal) or hypoxia/reoxygenation (H/R). QRT-PCR analyses for MIAT were then performed. Carv inhibits the expression of MIAT in HCFs (**C**), human cardiomyocytes (**D**), rat cardiomyocytes (**E**), and mouse cardiomyocytes (**F**) subjected to H/R. Moreover, MIAT is upregulated in HCFs as well as human and rodent cardiomyocytes after H/R. Data are shown as the fold induction of expression normalized to GAPDH genes. *N* = 5–7 per group. Two-way ANOVA with Tukey’s multiple comparison test. ^*^P < 0.05, **P < 0.01, or ^***^P < 0.001 vs. DMSO. ^#^*P* < 0.05, ^##^*P* < 0.01, or ^###^*P* < 0.001 vs. normoxia. Data are presented as the mean ± SEM.

Carvedilol stimulates β-arr1-mediated β_1_AR or β_2_AR signaling [31, 32] and miR-150 expression in the heart [15]. We also showed that MIAT was a key negative regulator of carvedilol/β_1_AR/β-arr1-responsive miR-150 in the heart [14]. This led us to examine whether carvedilol regulates left ventricular MIAT in mice. Indeed, cardiac MIAT is significantly downregulated upon carvedilol stimulation for 3 days [D] (Fig. 1A) and 7D (see wild type [WT] in Fig. 1B) when compared to the vehicle control. We next tested whether β-arr1 signaling is required for inhibition of MIAT by carvedilol. We find that the carvedilol-mediated inhibition of MIAT occurring in WT mice is not observed in hearts from β-arr1 KO mice (Fig. 1B). Because carvedilol is a β-arr1-biased ligand for both β_1_AR and β_2_AR [31, 32], we then measured MIAT expression in hearts from β_1_AR KO or β_2_AR KO mice. We show that the carvedilol-mediated inhibition of MIAT seen in WT mice is blunted in hearts lacking β_1_AR (Fig. 1B). We also observe that basal cardiac MIAT expression is not significantly different in β-arr1 KO mice (*P* = 0.929) or β_1_AR KO mice (*P* = 0.196) compared with WT controls. These findings suggest that carvedilol inhibits MIAT function through β-arr1–mediated β_1_AR signaling.

### Carvedilol inhibits the expression of MIAT in primary adult human cardiac fibroblasts as well as human and rodent CMs

Because MIAT is downregulated in mouse hearts treated with carvedilol, we next tested whether MIAT is similarly regulated in primary adult human cardiac fibroblasts (HCFs) as well as human and rodent CMs treated with carvedilol. Concurrent with miR-150 downregulation [14], we first find that MIAT is increased in HCFs (Fig. 1C), human CMs (Fig. 1D), and rodent CMs (Fig. 1E, F) after H/R that is consistent with our in vivo results in post-MI hearts and isolated CFs and CMs from MI mice [14]. We also observe that MIAT is downregulated in HCFs (Fig. 1C), human CMs (Fig. 1D), and rodent CMs (Fig. 1E, F) subjected to H/R conditions after carvedilol treatment, concurrent with miR-150 upregulation [14]. These data indicate that MIAT is also sensitive to carvedilol in HCFs as well as human and rodent CMs.

### BACH2, a transcriptional repressor and β-arr1-interacting protein, binds to conserved sites in the MIAT promoter, repressing MIAT transcription

**Fig. 2:**
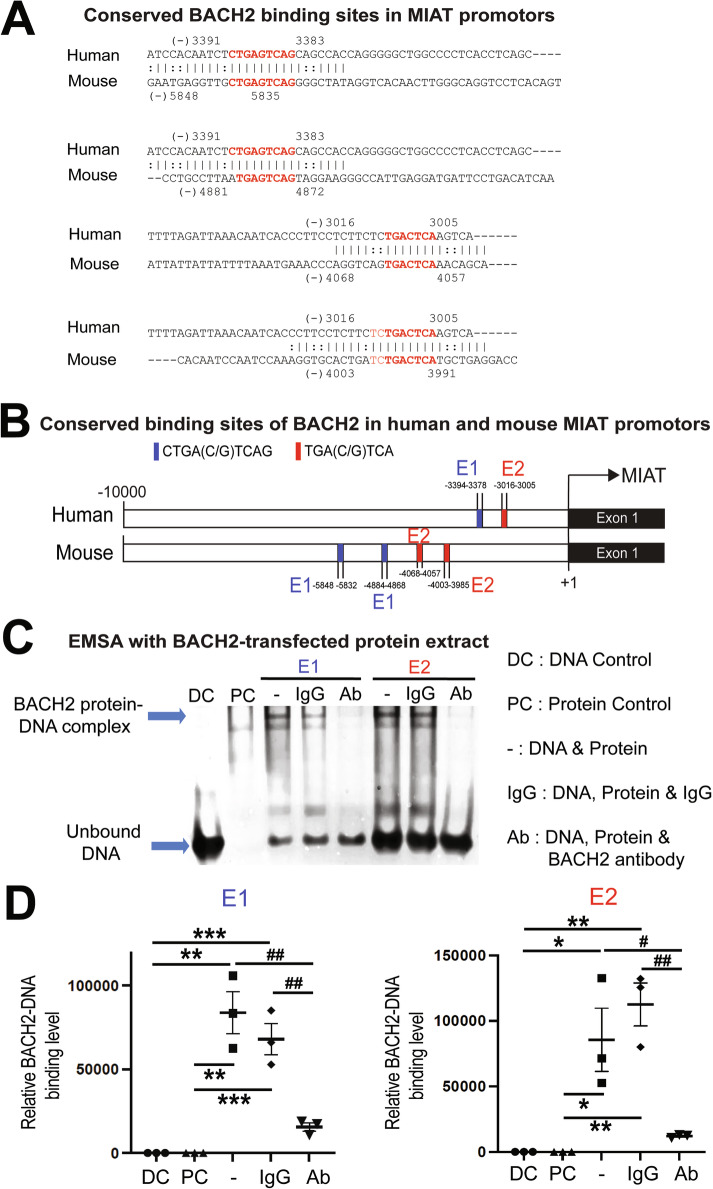
BACH2 binds to conserved regions of the MIAT promoter. **A** Sequence homology and conserved BACH2 binding regions (E1 and E2) within human and mouse MIAT promoters. Potential BACH2 binding sites are shown as red fonts. **B** Potential BACH2-binding sites conserved between human and mouse MIAT promoters. The binding site is shown only when the recognition sequence is found in both human and mouse MIAT promoters. **C**, **D** Electrophoretic mobility shift assays (EMSAs) were performed with oligonucleotides containing two conserved BACH2-binding regions (E1 and E2) in the human promoter. DNA-BACH2 protein complexes are markedly decreased by the addition of BACH2 antibodies, whereas the formation of specific BACH2-DNA complexes (shown with top blue arrow in **C**) is not inhibited by IgG antibodies. One-way ANOVA with Tukey’s multiple comparison test. ^*^P < 0.05, ^**^P < 0.01, or ^***^P < 0.001 vs. control: DC or PC. ^#^*P* < 0.05 or ^##^*P* < 0.01 vs. - or IgG.

We next investigated how carvedilol via β_1_AR/β-arr1 signaling inhibits MIAT expression. Because β-arr1 (not β-arr2) is known to translocate into nucleus and to regulate transcription, and β-arr1 had no DNA-binding domain [30], we hypothesized that carvedilol stimulation of β_1_AR promotes interaction between β-arr1 and a transcriptional repressor to inhibit cardiac MIAT transcription. A proteomic analysis reported the global cellular interactions of β-arr1 [33]. Among identified β-arr1-interacting proteins, we focus on transcription factors and detect conserved binding sites for BACH2 in human and mouse MIAT promoters (Figs. S1 and 2A, and Table S1). To determine whether BACH2 binds conserved sites in the MIAT promoter, we synthesized two double-stranded oligonucleotides corresponding to two human promoter regions (E1 and E2 in Fig. 2B). Electrophoretic mobility shift assays (EMSAs) were performed with these oligonucleotides and nuclear protein extracts from BACH2-transfected human CMs. BACH2 forms specific DNA-protein complexes, which are markedly inhibited by the addition of BACH2 antibodies. In contrast, the formation of BACH2-DNA complexes is not inhibited by IgG antibodies (Fig. 2C, D). These results support that BACH2 binds to conserved regions of the MIAT promoter.

**Fig. 3:**
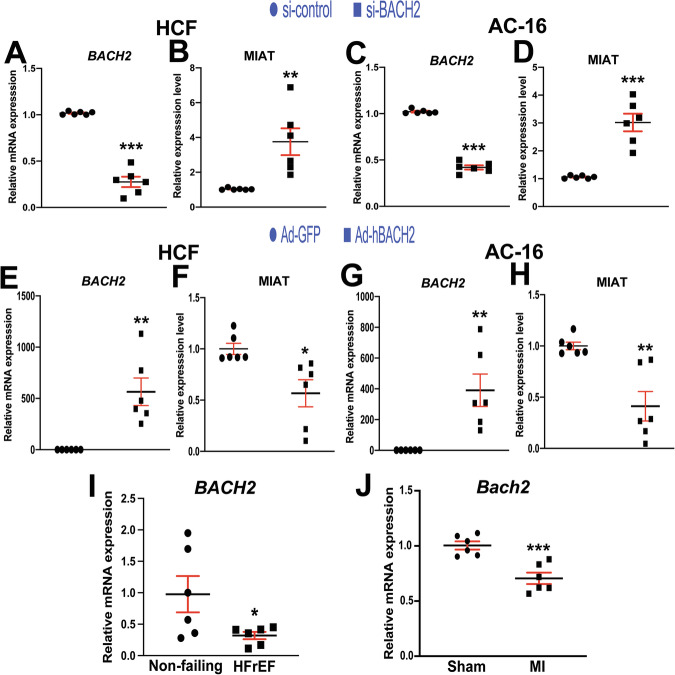
MIAT is inhibited by BACH2 in adult human cardiac fibroblasts and human cardiomyocytes, and cardiac BACH2 genes are downregulated in failing human and mouse hearts. Primary adult human cardiac fibroblasts (HCFs) (**A**, **B**) and AC-16 cardiomyocytes (**C**, **D**) were transfected with control scramble siRNA (si-control) or *BACH2* siRNA (si-BACH2). HCFs (**E**, **F**) and AC-16 cardiomyocytes (**G**, **H**) were infected with control adenovirus (Ad-GFP) or adenovirus expressing human BACH2 (Ad-hBACH2). QRT-PCR analyses for *BACH2* (**A**, **C**, **E**, **G**) or MIAT (**B**, **D**, **F**, **H**) were then performed to check their expression after the indicated transfection or infection. Data were normalized to *GAPDH* and are expressed relative to controls. *N* = 6 per group. Unpaired 2-tailed *t*-test. ^*^P < 0.01, ^**^P < 0.05, or ^***^P < 0.001 vs. control: either si-control or Ad-GFP control. Data are presented as the mean ± SEM. **I** QRT-PCR expression analysis of *BACH2* in left ventricles from patients with heart failure with reduced ejection fraction (HFrEF) relative to non-failing heart tissues. *N* = 6 per group. Unpaired 2-tailed *t*-test. ^*^P < 0.01 vs. non-failing control. **J** QRT-PCR expression analysis of *Bach2* in infarcted area at 2 weeks after MI relative to sham controls. *N* = 6 per group. Unpaired 2-tailed *t*-test. ^***^P < 0.001 vs. sham control. Data are presented as the mean ± SEM.

Although a prior proteomic analysis showed that BACH2 interacted with β-arr1 (not β-arr2) only after AngII treatment in HEK293 cells overexpressing angiotensin type 1 receptor (AT1R) [33], it remains to be determined whether BACH2 interacts with β-arr1 in human cells overexpressing β_1_AR after carvedilol stimulation. We thus performed co-immunoprecipitation (co-IP) experiments with the nuclear lysates of HEK293 cells overexpressing β_1_AR (β_1_AR stable cells) transiently overexpressing tagged plasmids with and without carvedilol treatment. We observe that carvedilol induces an association of β-arr1 with BACH2 in the nuclear lysates of β_1_AR stable cells (Fig. S2). Our data suggest that carvedilol stimulation of the β_1_AR promotes the interaction between β-arr1 and BACH2 to inhibit MIAT transcription in the nucleus. Indeed, our loss- and gain-of-function studies next reveal that MIAT is significantly repressed by *BACH2* in HCFs and human CMs (Fig. 3A−H). We also find for the first time that left ventricular *BACH2* is downregulated in patients with HFrEF (Fig. 3I) concurrent with MIAT upregulation [19]. Our human data on cardiac downregulation of *BACH2* agree with our mouse data post-MI (Fig. 3J). Taken together, we here report a novel axis among carvedilol, β_1_AR/β-arr1 signaling, a transcription factor BACH2, and a lncRNA MIAT. Our findings indicate that a nuclear β-arr1/BACH2 complex, which is induced by carvedilol-mediated β_1_AR signaling, binds to MIAT promoter and inhibits MIAT transcription.

### Carvedilol upregulates cardiac Bach2 in a β_1_AR– and β-arr1–dependent manner

**Fig. 4:**
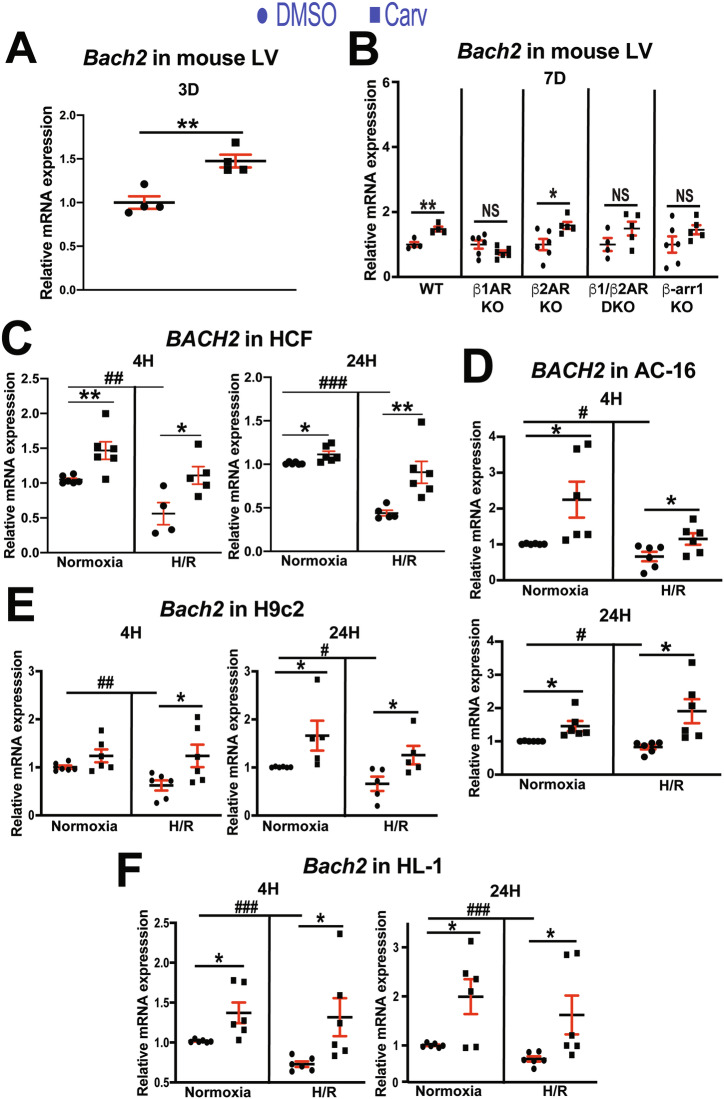
BACH2 genes are upregulated in mouse left ventricles, human cardiac fibroblasts, and human and rodent cardiomyocytes by carvedilol. *Bach2* expression was detected by QRT-PCR in left ventricles from adult mice stimulated with carvedilol (Carv: 19 mg/kg per day) or vehicle for 3 day (**A**) or 7 days (**B**). *N* = 4–6. Data are shown as fold induction of *Bach2* expression normalized to *Gapdh*. Unpaired 2-tailed *t*-test. ^*^P < 0.05 or ^**^P < 0.01 vs. DMSO. NS: not significant. **C**–**F** Primary adult human CFs (HCFs) as well as AC-16, H9c2, and HL-1 cardiomyocytes were treated with 1 μM Carv for 4 or 24 h (H) and subjected to either normoxia (basal) or hypoxia/reoxygenation (H/R). QRT-PCR analyses for the expression of BACH2 genes were then performed. Carv activates the expression of BACH2 genes in HCFs (**C**), AC-16 cells (**D**), H9c2 cells (**E**), and HL-1 cells (**F**) subjected to H/R. Moreover, the expression of BACH2 genes is downregulated in HCFs as well as AC-16, H9c2, and HL-1 cardiomyocytes after H/R. Data are shown as the fold induction of expression normalized to GAPDH genes. *N* = 4–6 per group. Two-way ANOVA with Tukey’s multiple comparison test. ^*^P < 0.05 or ^**^P < 0.01 vs. DMSO. ^#^*P* < 0.05, ^##^*P* < 0.01, or ^###^*P* < 0.001 vs. normoxia^.^ Data are presented as the mean ± SEM.

Based on our novel data showing (I) β-arr1’s nuclear interaction with BACH2 induced by carvedilol-mediated β_1_AR signaling (Fig. S2) and (II) BACH2’s binding to human MIAT promoter (Fig. 2C, D), we next tested whether *Bach2* and MIAT are inversely regulated in mouse left ventricles treated with carvedilol. We find that left ventricular *Bach2* is significantly upregulated upon carvedilol stimulation for 3D (Fig. 4A) and 7D (see WT in Fig. 4B) concurrent with MIAT downregulation (Fig. 1A, B). Our in vivo protein analysis also reveals that left ventricular levels of BACH2 are significantly elevated following carvedilol stimulation for 7D compared to DMSO-treated controls (Fig. S3). Because β-arr1 was localized to the nucleus, associated with transcription cofactors such as p300 and CREB at the promoters of target genes [30], and interacted with BACH2 [33], we next assessed whether β-arr1 is required for activation of cardiac *Bach2* by carvedilol. The carvedilol-mediated activation of cardiac *Bach2* that occurs in WT mice is not observed in β-arr1 KO mice (Fig. 4B). We then show that the carvedilol-mediated activation of *Bach2* seen in WT mice is blunted in hearts lacking β_1_AR (Fig. 4B). Our data also reveal that the basal expression of *Bach2* is not significantly changed in hearts from β-arr1 KO mice (*P* = 0.159) or β_1_AR KO mice (*P* = 0.089) when compared with WT controls. These data suggest that carvedilol stimulation of β_1_AR promotes interaction between β-arr1 and p300 or CREB at the promoter of *Bach2* to activate cardiac *Bach2* transcription.

### Concurrent with MIAT downregulation, carvedilol activates BACH2 expression in HCFs as well as human and rodent CMs

Because *Bach2* is upregulated in mouse hearts treated with carvedilol, we next tested whether the expression levels of BACH2 and MIAT are inversely regulated in HCFs as well as human and rodent CMs treated with carvedilol. Concurrent with MIAT upregulation (Fig. 1C−F), we first find that BACH2 expression is decreased in HCFs as well as human and rodent CMs after H/R (Fig. 4C−F) that is consistent with our results in mouse hearts post-MI and in failing human hearts (Fig. 3I, J). We also observe that the expression of BACH2 gene is increased in HCFs as well as human and rodent CMs subjected to H/R conditions after carvedilol (Fig. 4C−F), concurrent with MIAT downregulation (Fig. 1C−F). These data suggest that the expression of BACH2 gene is also sensitive to carvedilol in CFs and CMs.

### BACH2 represses HCF activation

**Fig. 5:**
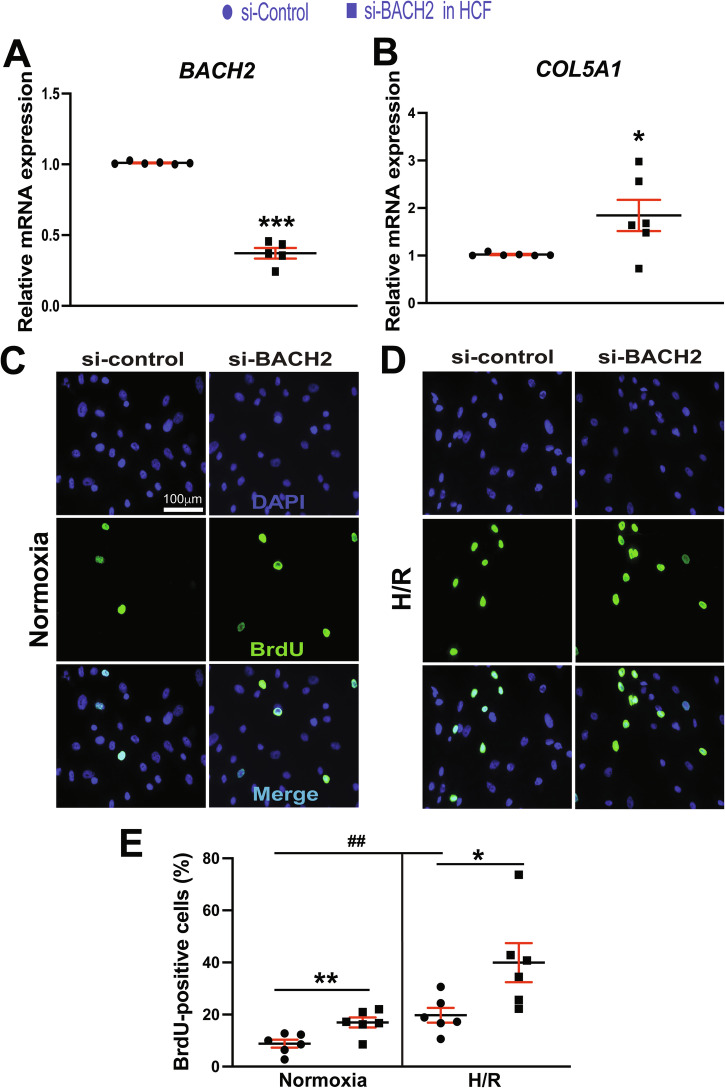
*BACH2* inhibits the expression of profibrotic *COL5A1* and suppresses proliferation of primary adult human cardiac fibroblasts. Primary adult human cardiac fibroblasts (HCFs) were transfected with control scramble siRNA (si-control) or *BACH2* siRNA (si-BACH2). QRT-PCR analyses for *BACH2* (**A**) or *COL5A1* (**B**) were then performed to check their expression after the indicated transfection. Data were normalized to *GAPDH* and are expressed relative to controls. *N* = 5–6 per group. Unpaired 2-tailed *t*-test. *P < 0.01 or ***P < 0.001 vs. si-control. **C**–**E** HCFs, which were transfected with si-control or si-BACH2, were subjected to hypoxia/reoxygenation (H/R). Bromodeoxyuridine (BrdU) assays were then performed. The percentage of proliferating nuclei (green) was calculated by normalizing to the total DAPI-stained nuclei (blue). *N* = 6 per group. Two-way ANOVA with Tukey’s multiple comparison test. ^*^P < 0.05 or ^**^P < 0.01 vs. si-control. ^##^*P* < 0.01 vs. normoxia. Data are presented as the mean ± SEM.

**Fig. 6:**
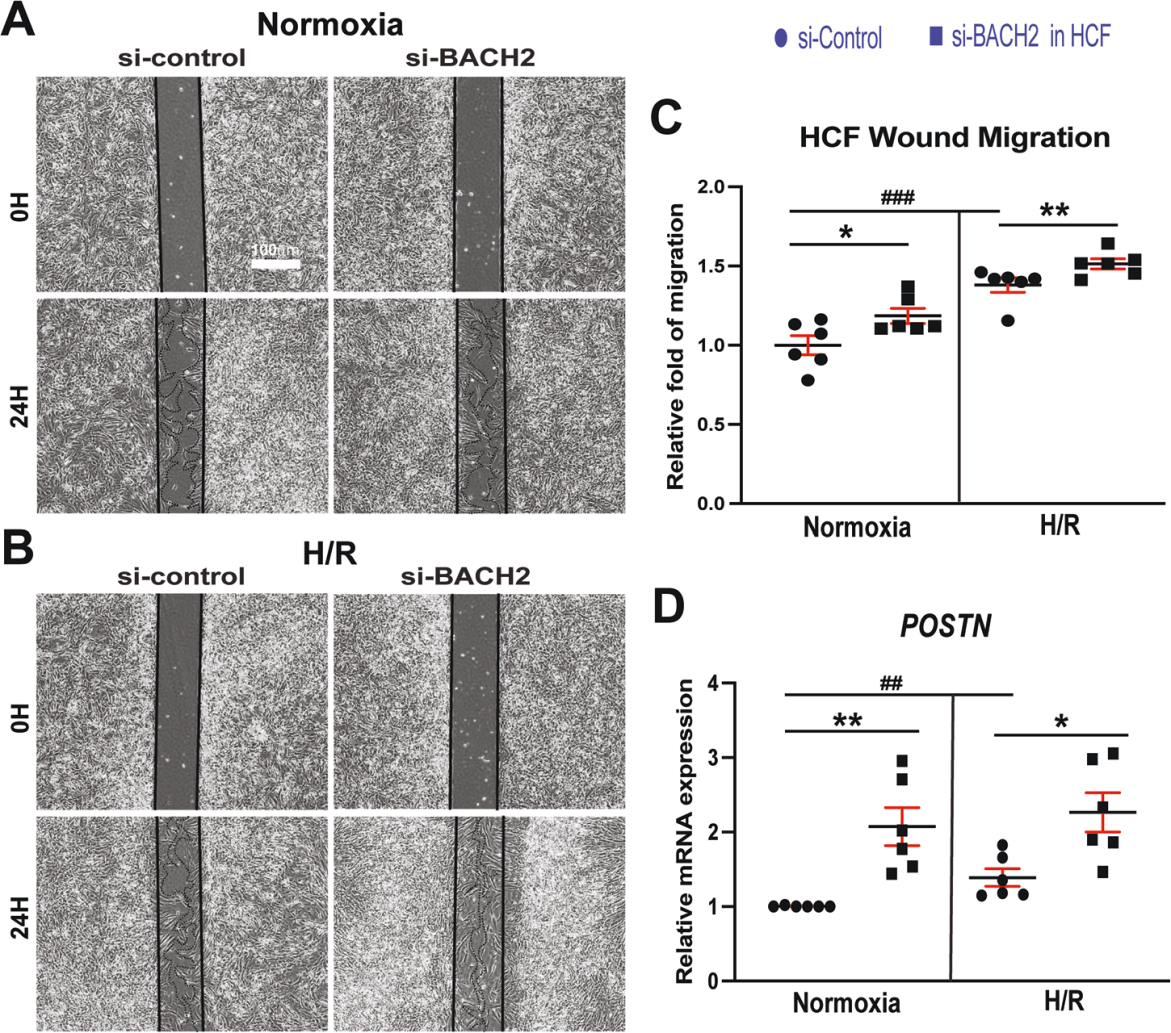
*BACH2* suppresses migration of primary adult human cardiac fibroblasts and inhibits the expression of profibrotic *POSTN.* **A–C** HCFs, which were transfected with si-control or si-BACH2, were subjected to H/R. Scratch migration assays were then performed. Initial open areas (0 h; H) were measured to serve as total open area, and the % of open area after 24 H was calculated to quantify migratory potential. **D** HCFs were transfected as indicated above. QRT-PCR analyses of *POSTN* were then performed. Data are shown as the fold induction of gene expression normalized to *GAPDH*. *N* = 6 per group. Two-way ANOVA with Tukey’s multiple comparison test. ^*^P < 0.05 or ^**^P < 0.01 vs. si-control. ^##^*P* < 0.01 or ^###^*P* < 0.001 vs. normoxia. Data are presented as the mean ± SEM.

MIAT was shown to promote CF activation and cardiac fibrosis post-MI [23]. We report here that BACH2 binds to conserved sites in the MIAT promoter, thus repressing MIAT expression (Figs. 2 and 3). We also demonstrate that concurrent with MIAT upregulation [14, 19], cardiac expression of BACH2 gene is downregulated in patients with HF and MI mice (Fig. 3I, J). Moreover, MIAT and *BACH2* are inversely regulated in HCFs treated with carvedilol and in HCFs subjected to H/R conditions (Figs. 1C and 4C). However, the role of the novel upstream regulator of MIAT, *BACH2* in HCF activation remains to be defined. Our loss-of-function studies uncover that compared to controls, *BACH2* knockdown in HCFs activates the expression of profibrotic *COL5A1* (Fig. 5A, B). We also show that decreasing *BACH2* expression promotes proliferation (Fig. 5C−E) and migration (Fig. 6A−C) as well as the expression of profibrotic *POSTN* (Fig. 6D) in HCFs subjected to normoxia and H/R conditions. Conversely, we observe that BACH2 overexpression decreases the expression of profibrotic *POSTN* and *COL3A1* in HCFs (Fig. S4). Our data thus indicate that *BACH2* functions as a negative regulator of HCF activation.

### BACH2 acts as a negative regulator of apoptosis in human and mouse CMs

**Fig. 7:**
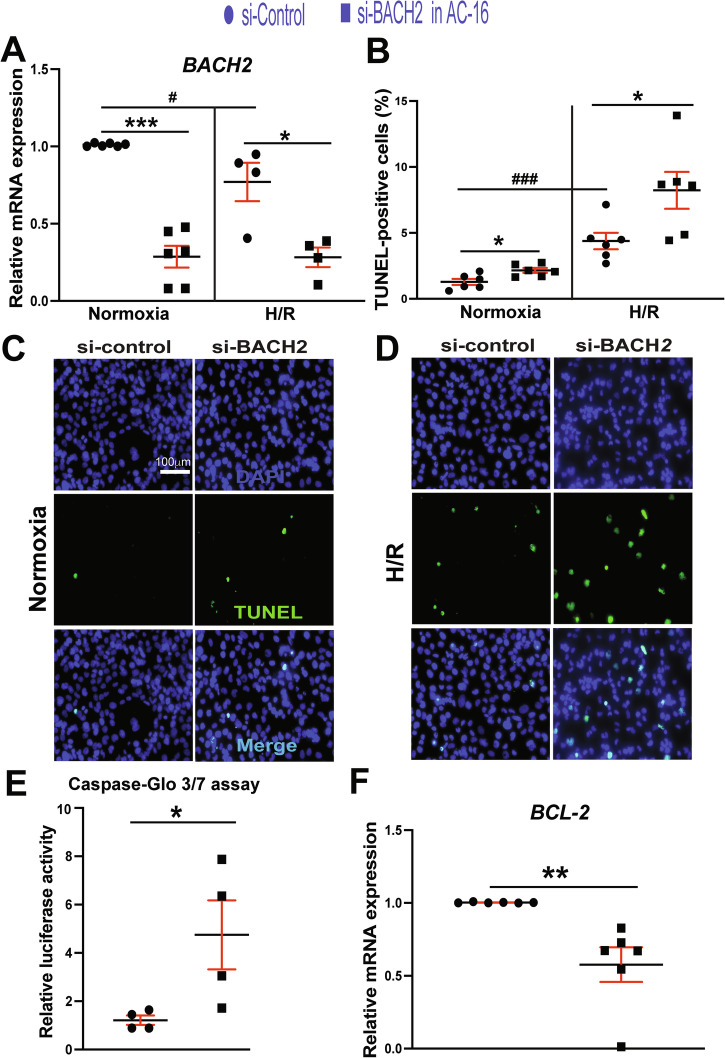
*BACH2* is downregulated in human cardiomyocytes after H/R and suppresses apoptosis in human cardiomyocytes. **A–D** AC16 cells, which were transfected with si-Control or si-*BACH2*, were subjected to H/R. QRT-PCR for *BACH2* was conducted to check the knockdown efficiency. Data were normalized to *GAPDH* and expressed relative to controls (**A**). TUNEL assays were also conducted in both normoxic and H/R conditions. Scale bar = 100 μm. The percentage of apoptotic nuclei (green) was calculated after the normalization of total nuclei (blue) (**B–D**). *N* = 4–6 per group. Two-way ANOVA with Tukey’s multiple comparison test. ^*^P < 0.05 or ^***^P < 0.001 vs. si-Control. ^#^*P* < 0.05 or ^###^*P* < 0.001 vs. normoxia. **E** AC16 cells were transfected with si-Control or si-*BACH2* and processed for Caspase-Glo 3/7 luciferase assays. *N* = 4/group. Unpaired two-tailed *t*-test. ^*^P < 0.05 vs. si-Control. **F** QRT-PCR expression analysis of anti-apoptotic *BCL2* in AC16 cells transfected with 2 different groups as indicated. *BCL2* expression compared to *GAPDH* was calculated using 2^−ΔΔCt^, and data are presented as fold induction of *BCL2* expression levels normalized to si-Control. *N* = 6. Unpaired two-tailed *t*-test. **P < 0.01 vs. si-Control. All data are shown as mean ± SEM.

*Bach2* knockdown in CMs worsened TAC-induced HF [6] and aggravated diabetic HF [8]. *Bach2* was also reduced in CMs treated with ISO [6]. Moreover, MIAT knockdown inhibited CM apoptosis against HG or H/R [21, 22]. Because carvedilol upregulates BACH2 gene in human and rodent CMs concurrent with MIAT downregulation (Figs. 1D−F and 4D−F), we next tested whether BACH2 regulates CM apoptosis. We first find that *BACH2* knockdown in human CMs increases TUNEL-positive nuclei in basal and H/R conditions (Fig. 7A−D). In agreement with this data, we also observe that human CMs with *BACH2* knockdown exhibit enhanced Caspase 3/7 luciferase activity and suppressed expression of anti-apoptotic *BCL-2* (Fig. 7E, F). Conversely, our gain-of-function studies in human CMs also show that *BACH2* overexpression results in increased expression of anti-apoptotic *BCL-2* and decreased expression of apoptotic *KLF-13* (Fig. S5A−C). Overexpression of *BACH2* suppresses human CM apoptosis in basal and H/R conditions (Fig. S5D−F). Last, we observe that knocking down *Bach2* in mouse CMs increases apoptosis in basal and H/R conditions (Fig. S6A−D). These TUNEL data agree with our Real-time Quantitative Reverse Transcription Polymerase Chain Reaction (QRT-PCR) data, showing that *Bach2* knockdown causes the increased expression of apoptotic *Bak1*, *P2x7r*, and *Ing4* (Fig. S6E−G). Thus, these CM data indicate that BACH2 is a critical negative regulator of CM apoptosis.

## Discussion

Here, we identify the BACH2–MIAT interaction as a novel regulatory mechanism underlying β_1_AR/β-arr1 protective signaling in the context of ischemic injury. Our data show that carvedilol downregulates MIAT while concurrently inducing BACH2 expression in cultured CFs and CMs as well as mouse left ventricles in a β_1_AR/β-arr1-dependent manner. Mechanistically, we show that BACH2—a β-arr1-interacting transcriptional repressor—binds to conserved sites in the MIAT promoter, thereby suppressing the expression of profibrotic and proapoptotic MIAT, inhibiting HCF activation, and reducing apoptosis in both human and mouse CMs. We previously reported using MIAT mouse models that systemic or cardiac MIAT promoted maladaptive post-MI remodeling by upregulating profibrotic and proapoptotic gene programs [14, 16]. In line with these findings, our current study demonstrates that overexpression of BACH2 in CFs and CMs represses MIAT and improves cellular function after ischemic stress, whereas BACH2 deficiency leads to increased MIAT expression, heightened CF activation, and enhanced CM apoptosis.

Nuclear functions of β-arr1 were previously reported. β-Arr1 serves as a nuclear transcriptional regulator of endothelin type A receptor–mediated β-catenin signaling [34] and acts as a key modulator of polycomb group proteins following δ-opioid receptor stimulation [29]. β-Arr–mediated β_1_AR signaling has been shown to confer cardioprotective effects [35]. We previously reported that carvedilol-induced β_1_AR/β-arr1 signaling upregulated miR-150 [15]. Although multiple studies have investigated the role of noncoding RNAs (ncRNAs) in HF and uncovered important ncRNA-mediated regulatory mechanisms [36–38], to our knowledge, no studies have examined whether β_1_AR/β-arr-mediated signaling regulates lncRNA activity in the heart. In earlier work, we established direct in vivo functional links between MIAT and miR-150, as well as between cardiac β_1_AR/β-arr signaling and miR-150, in the setting of HF [14, 39]. Our current data (Figs. 1B and 4B) further demonstrate that β_1_AR/β-arr1 signaling is essential for carvedilol-mediated MIAT repression and for the activation of BACH2, which is mechanistically linked to protection of both CFs and CMs. We also find that cardiac BACH2 expression is downregulated in patients with HF and MI mice (Fig. 3I, J), in parallel with MIAT upregulation [14, 19]. Taken together with the findings presented here, our results suggest that BACH2 may function as a critical downstream effector of β_1_AR/β-arr1 signaling pathways, mediating its protective effects. Specifically, our study identifies a novel β_1_AR/β-arr1-driven regulatory pathway in which BACH2 activation contributes to favorable cardiac remodeling by suppressing CF activation and CM apoptosis through inhibition of maladaptive lncRNAs, including MIAT.

MIAT was reported to activate the proapoptotic transcription factor EGR2, thereby promoting CM apoptosis and contributing cardiac dysfunction [40]. Additionally, MIAT was identified as a profibrotic lncRNA in post-infarct hearts by increasing FURIN, an activator of TGF-β1 [23]. In our previous study, we demonstrated that MIAT promoted cardiac fibrosis following ischemic stress in part by antagonizing the antifibrotic effects of miR-150 on HOXA4 through its competing endogenous RNA activity [14]. We also found that CM-derived MIAT exacerbated the response to MI by suppressing miR-150, a key mediator of CM survival and antifibrotic signaling [41, 42], while simultaneously upregulating multiple proapoptotic and profibrotic genes [16]. Despite these advances, the upstream regulatory pathways governing the proapoptotic and profibrotic actions of MIAT remained incompletely understood. To address this gap, our current study identifies a novel β_1_AR/β-arr1 protective signaling axis involving BACH2 as a key transcriptional regulation of MIAT, revealing its defining role in modulating CM apoptosis and CF activation.

BACH2, a member of the BACH family of transcription factors, is part of the understudied Druggable Proteome. Prior studies associated BACH2 with genetic susceptibility to type 1 diabetes [4, 5] and various autoimmune disorders [43]. *Bach2* KO mice exhibited severe postnatal weight loss and high mortality [44]. In pancreatic β-cells, BACH2 suppressed apoptosis by regulating the mitochondrial apoptotic pathway [45]. Intriguingly, BACH2 was significantly downregulated in human hypertrophic hearts, in mouse hearts following TAC, and in CMs treated with ISO, compared to controls [6]. Both mouse and neonatal rat ventricular cardiomyocyte (NRVC) studies showed that BACH2 overexpression protected against TAC-induced HF and attenuated ISO-induced CM hypertrophy. Conversely, systemic or CM-specific knockdown of *Bach2* worsened TAC-induced HF in mice, and *Bach2* deficiency exacerbated ISO-triggered hypertrophy in NRVCs [6]. Mechanistically, BACH2 was shown to repress *Akap6*, a key scaffold protein involved in cardiac hypertrophy and HF, by directly binding to its promoter [6].

Consistent with prior reports showing that myricetin had beneficial effects in MI, ischemia/reperfusion (I/R) injury, and ISO-induced HF [7], myricetin ameliorated TAC-induced HF in part by upregulating BACH2 [6]. The same group further demonstrated that BACH2 was negatively correlated with diabetes-induced myocardial injury in humans. CM-specific BACH2 overexpression attenuated diabetic cardiomyopathy in mice, whereas conditional KO of *Bach2* in CMs worsened diabetic HF [8]. Mechanistically, BACH2 served as a convergent hub for cannabinoid receptor 2-mediated repression of necroptosis-related genes in HG-treated mouse CMs [8]. Although BACH2 was described as an antihypertrophic and antidiabetic factor in CMs, its role in CF activation and CM apoptosis under ischemic stress has not been explored. The present study addresses how BACH2 is regulated and can modulate CF activation and CM apoptosis. We show that cardiac BACH2 is upregulated by the β-blocker carvedilol via β_1_AR/β-arr1 signaling, coinciding with carvedilol-mediated downregulation of MIAT (Figs. 1B and 4B). Furthermore, our mechanistic data demonstrate that BACH2 forms a nuclear complex with β-arr1 and binds to conserved regions within the MIAT promoter (Figs. 2 and S2). Finally, our primary HCF as well as human and rodent CM studies provide compelling evidence that BACH2 inhibits the profibrotic and proapoptotic activity of MIAT, thereby suppressing CF activation and CM apoptosis (Figs. 3, 5–7 and S4–S6).

Circulating MIAT, presumably released by CFs and CMs, was identified as a superior biomarker for HF compared to clinically established markers such as BNP and cTnT [20, 46]. Our current findings in mouse hearts as well as in CFs and CMs support the concept that targeting the BACH2/MIAT axis may represent a promising therapeutic strategy for HF. Given our results demonstrating that the profibrotic and proapoptotic lncRNA MIAT is a novel direct target of β_1_AR/β-arr1-responsive BACH2 in both CFs and CMs, elevated circulating MIAT levels in patients with HF could potentially serve as a biomarker to guide current and future treatment strategies targeting the β_1_AR/β-arr1/BACH2/MIAT pathway.

### Limitations

Our current data indicate that β-arr1 and BACH2 form a nuclear complex that binds to conserved DNA elements within the MIAT promoter. To further validate the proposed link among MIAT, the BACH2/β-arr1 complex, and carvedilol-mediated β_1_AR signaling as well as to confirm specificity and in vivo binding, additional studies such as EMSAs with competitor controls, chromatin immunoprecipitation, cleavage under targets and tagmentation, and luciferase reporter assays will be required. Although we demonstrate that β_1_AR/β-arr1-responsive BACH2 represses CF activation and CM apoptosis by inhibiting transcription of the profibrotic and proapoptotic lncRNA MIAT, a more complete understanding of the functional interplay among β_1_AR/β-arr1 signaling, BACH2, and MIAT as a novel regulatory mechanism in maladaptive CM and CF responses as well as overall cardiac pathology also remains to be established, though such investigation lies beyond the scope of the current study. In addition, it remains possible that BACH2 or MIAT expression in cardiac endothelial and inflammatory cells also contributes significantly to maladaptive cardiac remodeling. Future studies employing conditional, cell–type–specific mouse models are therefore warranted to elucidate the roles of BACH2 and MIAT in a cell-specific context. Finally, understanding whether the BACH2/MIAT axis exerts its effects through paracrine signaling between cell types or acts via autocrine mechanisms within individual cell populations will be critical.

## Conclusions

Our mouse studies using various KO models demonstrate that β_1_AR/β-arr1 protective signaling regulates the BACH2/MIAT axis. Complementary findings in CFs and CMs further indicate that BACH2 confers protective effects, at least in part, by repressing transcription of fibrotic and apoptotic MIAT, thereby mitigating CF activation and CM apoptosis. Given that downregulation of BACH2 or upregulation of MIAT is implicated in multiple forms of heart disease [6, 8, 19, 20], their roles in cardiac remodeling may extend across a range of pathological stress conditions. Accordingly, therapeutic strategies aimed at enhancing BACH2 expression or suppressing MIAT, such as BACH2 overexpression, carvedilol administration, or antisense oligonucleotide–mediated MIAT knockdown, may offer promising adjunctive approaches to attenuate CF activation and CM apoptosis, ultimately improving outcomes in HF.

## Materials and methods

### Knockout mouse study and carvedilol infusion

We employed 8 to 16-week-old WT, β_1_AR KO, β_2_AR KO, β_1_/β_2_AR double KO, and β-arr1 KO mice. Carvedilol (Sigma-Aldrich) was dissolved in dimethyl sulfoxide (DMSO), and micro-osmotic pumps (Alzet model 2001; DURECT Corporation) were then used to deliver carvedilol in WT, β_1_AR KO, β_2_AR KO, β_1_/β_2_AR double KO, and β-arr1 KO mice at the rate of 19 mg/kg/day for 3 or 7 days. In control mice, 10% DMSO was administrated as a vehicle. All mouse lines receiving carvedilol displayed no left ventricular dysfunction, and genotypic verification of these KO mice has been described previously [35, 47, 48]. Mice were maintained on a C57BL/6J background, and WT littermates were used as controls. Left ventricular tissues were then snap-frozen in the liquid nitrogen for subsequent analyses as described previously [15, 31].

### Minimization of pain and distress of mice

All efforts were made to minimize discomfort, distress, pain, and injury. Animals were handled carefully and humanely prior to anesthesia by approved methods. To perform MI surgery on the heart, adult mice were anesthetized using isoflurane (1–4%, inhalant). The topical local analgesia drug, bupivacaine (a few small drops of 0.75–1%), was administered at the time of surgery. Sustained-release meloxicam (4–5 mg/kg, subcutaneous injection) and extended-release buprenorphine (3.25 mg/kg, Ethiqa XR: extended-release formulation, subcutaneous injection) were administered once to provide up to 72 h of systemic analgesia. The animals were observed for the pinch toe reflex during the surgery. Following the surgery, the mice were monitored until they regained consciousness. Post-operative care included monitoring every 15–30 min following the surgery for 2–3 h and then daily until the study endpoint for signs of distress, including difficulty with breathing, grooming, defecation, eating, and mobility.

### Human heart samples

Left ventricular samples of failing human hearts were collected from ischemic cardiomyopathy and non-ischemic cardiomyopathy patients, who were hospitalized with HFrEF and underwent orthotopic cardiac transplantation as previously described [49]. Left ventricular tissues were dissected and snap-frozen in liquid nitrogen. The frozen samples were then stored in the specimen storage facility at the Indiana Clinical and Translational Sciences Institute located at Indiana University. Non-failing left ventricular tissues were obtained from donor hearts not suitable for transplantation, and they were collected and stored in the same manner. Demographic characteristics of these left ventricular tissue samples are provided in Table S2.

### Ethics committee approval

The animal experiments conducted as a part of this study complied with the Guidelines for the Care and Use of Laboratory Animals published by the US National Institutes of Health. Mice were euthanized by thoracotomy under 1–4% inhaled isoflurane. All experiments with mice were performed according to the protocols approved by the Institutional Animal Care and Use Committee at the Indiana University School of Medicine (approval reference #24139). Eight to sixteen-week-old C57BL/6J mice of both sexes were used for this study. Genotype- and sex-matched mice were randomly assigned to experimental groups to mitigate the cage effect. Operators were blinded to mouse genotypes until the end of the analysis. All the procedures involving human samples were conformed to the principles outlined in the Declaration of Helsinki and the Guidelines for the Health Insurance Portability and Accountability Act (HIPAA), as well as approved by the Indiana University Institutional Review Board (approval reference #08-018). All participants provided informed written consent prior to inclusion in the study.

### Statistics

Data are presented as the mean ± standard error of the mean (SEM) from independent experiments with different biological samples per group. Triplicate experiments were performed for all biochemical and cell biology studies. The number of in vitro biological samples per group was 4–7. The number of human samples per group was 6. Based on our retrospective data for experimental assays, a total of 3–11 mice of both sexes per group are used in the analyses, which provides adequate statistical power. The sample size of used mice for the current study is determined using a valid statistical model with a one-tailed hypothesis and 80% power. All mice included in the study are analyzed. The exact sample size for each experimental group/condition is given as a number in the figure legend and table. To allow the direct evaluation of the distribution of the data, we present graphical data as scatter/dot plots. Normality was assessed with the Kolmogorov–Smirnov test. The following statistical tests were used: unpaired two-tailed *t*-test for comparisons between 2 groups, one-way analysis of variance (ANOVA) with Tukey’s post-hoc test for multiple pairwise comparisons, and two-way ANOVA with Tukey’s post-hoc test for comparisons between 2 groups with different treatments. A *P* value of <0.05 was considered statistically significant. *P* values are indicated as follows: * ^or #^*P* < 0.05; ** ^or ##^*P* < 0.01; and *** ^or ###^*P* < 0.001.

## Supplementary information

Clean version of supplementary information including texts, tables, and figures

Unedited Gels and Blots

## Data Availability

All data are included in the manuscript and Supplementary Information. The analytical methods and study materials will be made available to other researchers for the purposes of reproducing the results or replicating the procedures. Additional methods are provided in Supplementary Information.
