# Supplementary material for: BACH2 links β1-adrenergic receptor/β-arrestin1 signaling to MIAT to inhibit cardiac fibroblast activation and cardiomyocyte apoptosis
Source: Cell Death Discov. 2026 Feb 28;12:127. doi: 10.1038/s41420-026-02985-4 (PMC13039337; doi:10.1038/s41420-026-02985-4)
Supplement: Supplementary file 1 — Clean version of supplementary information including texts, tables, and figures [file 41420_2026_2985_MOESM1_ESM.pdf]

Supplementary Information

BACH2 links β_1-adrenergic receptor/β-arrestin1 signaling to MIAT to inhibit cardiac fibroblast activation and cardiomyocyte apoptosis

Moukette et al., Running Title: Establishing the BACH2/MIAT axis in ischemic stress

Bruno Moukette[1,¶], Jian-peng Teoh[1,‡], Waleed J. Hashmi[1], Satoshi Kawaguchi[1,§,†], Tatsuya Aonuma[1,†], Hamedane Moustapha[1], Steven S. Welc[1], Simon J. Conway[2], Suthat Liangpunsakul[3,5], Lei Yang[2], Ankit A. Desai[4], and Il-man Kim[1,2,4,*]

[1]Department of Anatomy, Cell Biology, and Physiology, [2]Herman B Wells Center for Pediatric Research, [3]Division of Gastroenterology and Hepatology, and [4]Krannert Cardiovascular Research Center, Indiana University School of Medicine, Indianapolis, IN, USA. [5]Roudebush Veterans Administration Medical Center, Indianapolis, IN, USA.

[¶]Present address: Internal Medicine Research Unit, Pfizer Inc., Cambridge, MA, USA.
[‡]Present address: Shenzhen Institute of Synthetic Biology, Shenzhen Institutes of Advanced Technology, Chinese Academy of Sciences, Shenzhen, China.
[§]Present address: Department of Emergency Medicine, Asahikawa Medical University, Asahikawa, Hokkaido, Japan.
[†]Present address: Division of Cardiology and Nephrology, Department of Internal Medicine, Asahikawa Medical University, Asahikawa, Hokkaido, Japan.

*Address for correspondence:
Il-man Kim, PhD
Associate Professor
Department of Anatomy, Cell Biology, and Physiology
Indiana University School of Medicine
635 Barnhill Drive, MS 346A, Indianapolis, IN 46202, USA
Email: ilkim@iu.edu and Phone: 317-278-2086

This Supplementary Information file includes:

- Supplementary Text
- Figures S1 to S6
- Tables S1 to S2
- Supplementary References

Other supporting materials for this manuscript include the following:

- Unedited Original Gel and Blot Images

Supplementary Materials and Methods

Mouse model of myocardial infarction

The animals were originated from breeding taking place in a barrier Animal Care Unit at Indiana University. Animals were housed in the institution's animal-care facility accredited by the Association for Assessment and Accreditation of Laboratory Animal Care International. All mice were kept in bedded and sterile microisolator caging with filtered cage tops. They were also provided with standard pelleted food and water ad libitum as well as maintained at the temperature- and humidity-controlled rooms on the 12-hour light and 12-hour dark cycle. Eight- to sixteen-week-old wild type (WT) mice were subjected to myocardial infarction (MI) as we published [1-5]. Briefly, mice were anesthetized using isoflurane (1–4%, inhalant) and placed on a heating pad. Mice were intubated and ventilated with oxygen using a PhysioSuite MouseVent^{TM} ventilator (Kent Scientific). The left anterior descending (LAD) coronary artery was visualized under a stereoscope and ligated by using an 8-0 nylon suture. Regional ischemia was confirmed by visual inspection for discoloration of the occluded distal myocardium. Sham-operated mice were undergone the same procedure without LAD occlusion. The topical local analgesia drug, bupivacaine (a few small drops of 0.75–1%) was administered at the time of surgery. Sustained-release meloxicam (4–5mg/kg, subcutaneous) and sustained-release buprenorphine (3.25 mg/kg Ethiqa XR; MWI Animal Health, subcutaneous) were also given once immediately before the surgery to provide up to 72 hours of systemic analgesia. The mice were observed for the pinch-toe reflex every 15 minutes during the surgery. Following the surgery, the mice were monitored until they regained consciousness. Post-operative care included monitoring every 15–30 minutes following the surgery for 2–3 hours and then daily until the study endpoint for signs of distress, including difficulty with breathing, grooming, defecation, eating, and mobility. We used responses to toe/skin pinch and heart rate for the optimal anesthesia and appropriate post-operative monitoring plan. Mice, whose pain could not be managed and who exhibited such symptoms of

distress, were euthanized immediately and humanely. Records detailing the procedures and pharmacological interventions given to the mice were maintained.

Primary adult human cardiac fibroblast culture

The primary adult human cardiac fibroblasts (HCFs) were purchased from PromoCell (C12375) and maintained according to the company's recommendation. The primary HCFs, which were isolated from the ventricles of the adult heart, display normal fibroblastic characteristics and cellular markers (*i.e.*, CD90-positive, smooth muscle α-actin-negative and slow muscle myosin-negative). We used multiple batches of HCFs with passage number of 3–5 to further ensure the specificity of used cells and normal growth pattern. The adult primary HCFs were cultured in PromoCell Fibroblast Growth Medium 3 (C-23025), which contains basic fibroblast growth factor (bFGF: 1ng/ml), recombinant human insulin (5ug/ml) and 10% fetal calf serum (FCS: 0.1ml/ml) in the supplement.

Cardiomyocyte culture

The immortalized mouse adult atria-derived cardiomyocyte (CM) HL-1 cell line obtained from Dr. Claycomb was maintained and used as previously described [6]. Rat embryonic ventricular CM H9c2 cells were used as we published [6, 7]. The AC16 adult human CM cell line was purchased from Sigma-Aldrich (SCC109) and maintained according to the company's recommendation. The cell line was established from primary cells by transfecting the SV40 T antigen, and it displayed normal CM characteristics and cellular markers. We used multiple batches of the cell lines subjected to 3–5 passages in culture. Both HL-1 and AC16 CM cell lines were authenticated by the supplier, and they have been monitored regularly for their authenticity and to be negative for mycoplasma contamination.

Transfection and adenovirus infection studies in cells

Using Lipofectamine™ 3000 reagent (Invitrogen) as previously described [5, 8], HCFs and CMs were transfected with ON-TARGETplus Non-targeting Negative Control siRNA (Horizon Discovery Ltd, D-001810-10-05), an ON-TARGETplus siRNA-SMARTpool targeting human *BACH2* (Horizon Discovery Ltd, L-009787-00-0005, ID: 60468), or an ON-TARGETplus siRNA-SMARTpool targeting mouse *Bach2* (Horizon Discovery Ltd, L-042963-01-0005, ID: 12014) for loss-of-function studies. For gain-of-function studies, HCFs and AC16 cells (8×10^6 cells) were infected at a multiplicity of infection of 0.2 infectious units/cell with an adenovirus containing an expression vector for human BACH2 (Ad-BACH2) (Vector Biolabs, ADV-229367). Ad-LacZ was used as a control. AC16 cells were transiently transfected with a CMV-BACH2 expression vector using Lipofectamine™ 3000 reagent (Invitrogen) for electrophoretic mobility shift assays. For immunoprecipitation studies, HEK-293 cells stably expressing β_1-adrenergic receptor (β_1AR stable cells) were transfected with HA-β-arrestin1 and FLAG-BACH2 expression vectors. All in vitro assays were performed 60–72 hours after transfection or infection when maximum knockdown efficiency was reached.

Carvedilol treatment in cells and an in vitro model of hypoxia/reoxygenation (H/R)

For carvedilol stimulation experiments, HCFs and CMs were stimulated with carvedilol (1 μM; Sigma-Aldrich) in serum-free media for 4–24 hours as described previously [7], and were then processed for hypoxia/reoxygenation as below. HCFs and CMs transfected as aforementioned or treated with carvedilol were incubated in an ischemia buffer that contained 118 mM NaCl, 24 mM NaH_2CO_3, 1 mM $NaHPO_4$, 2.5 mM $CaCl_2$, 1.2 mM $MgCl_2$, 20 mM sodium lactate, 16 mM KCl, and 10 mM 2-deoxyglucose (pH 6.2). HCFs were then placed in the hypoxic chamber (5% CO_2, 0.1% O_2) for 3 hours, followed by 18 hours of reperfusion-mimicking conditions (*i.e.*, replacing the ischemic buffer with normal cell medium under normoxic conditions) as described [5, 9]. Our previous study also validated this in vitro model, and we showed that the model caused significantly increased apoptosis in primary CFs [10]. Based on our initial optimization

experiments in HCFs with different time points (2–3 hours of hypoxia and 4–18 hours of reoxygenation), we chose 3 hours of hypoxia and 18 hours of reoxygenation when maximum increases in the expression of pro-fibrotic *POSTN* and *COL3A* were achieved, as compared to normoxia. CMs were placed in the hypoxic chamber (5% CO_2, 0.1% O_2) for 3 hours followed by replacing the ischemic buffer with a normal cell medium, and were incubated under normoxia conditions for 4 hours to complete the H/R protocol as described [5, 9]. Transfected or carvedilol-treated cells were then processed for Real-time Quantitative Reverse Transcription Polymerase Chain Reaction (QRT-PCR), BrdU proliferation, wound migration, immunoblotting, TUNEL staining, or caspase-Glo 3/7 assays as mentioned below.

RNA isolation and QRT-PCR

Total RNAs from left ventricles of human and mouse hearts, HCFs, human and rodent CMs, and the infarct area of mouse hearts were prepared using TRIzol Reagent (Thermo Fisher Scientific) and treated with RNase-free DNase I (Thermo Fisher Scientific) to remove genomic DNA as we published [11]. RNA quantity and quality were measured by the Synergy LX FA Multi-Mode Microplate Reader (BioTek Instruments). RNA integrity was assessed by standard denaturing agarose gel electrophoresis. Intact total RNA run on a denaturing gel had sharp 28S and 18S rRNA bands. The 28S rRNA band was approximately twice as intense as the 18S rRNA band, further confirming that the RNA was intact. We then used the Minimum Information for Publication of Quantitative Real-Time PCR Experiments (MIQE) Guidelines for QRT-PCR experiments.

cDNAs for genes or MIAT were generated using SuperScript IV reverse transcriptase (Thermo Fisher Scientific) and random hexamer primers. Expression of genes or MIAT was detected using TaqMan expression assays for mouse (*Bach2,* Mm00464379_m1; *Bak1,* Mm00432045_m1; *P2x7r*, Mm00440578_m1; *Ing4*, Mm00460097_m1; MIAT, Mm01196418_g1; and *Gapdh*, Mm99999915_g1 for an endogenous control), and human

(*BACH2,* Hs00935338_m1; *POSTN,* Hs01566750_m1; *COL3A1*, Hs00943809_m1; *COL5A1*, Hs00609133_m1; *BCL2*, Hs04986394_s1; *KLF13,* Hs00429818_m1; MIAT, Hs00402814_m1 and *GAPDH*, Hs02786624_g1 for an endogenous control). The following reaction components were used for each probe: 2 µL cDNA, 10 µL 2X TaqMan Universal PCR Master Mix (Thermo Fisher Scientific), 1 µL probe, and 7 µL nuclease-free water in a 20 µL total volume. QRT-PCR reactions were analyzed using a QuantStudio 3 Detection System (Thermo Fisher Scientific) as we published [12]. PCR reaction conditions were as follows: Step 1: 50 °C for 2 min, Step 2: 95 °C for 10 min, Step 3: 40 cycles of 95 °C for 15 seconds followed by 60 °C for 1 min. Expression compared to endogenous controls was calculated using $2^{-\Delta\Delta Ct}$, and expression levels were normalized to control.

Coimmunoprecipitation, Immunoblotting, and antibodies

Following stimulation with carvedilol for 16 hours in transfected β_1AR stable cells, nuclear protein extracts were prepared as described [13]. Prior to immunoprecipitation, 25 µl of lysates was aliquoted into a separate tube for protein estimation and analysis of nuclear input. Immunoprecipitation was carried out as previously described [14, 15]. The left ventricles from mice treated with carvedilol or vehicle control for 7 days were washed once with PBS and solubilized them in Nonidet P-40 lysis buffer. Immunoprecipitated or lysate samples were resolved by SDS-PAGE and transferred to PVDF membrane (Bio-Rad) for immunoblotting. FLAG or HA immunoblotting was carried out using monoclonal antibodies at dilutions of 1:5,000 each (Sigma-Aldrich) as previously described [8]. We purchased and used BACH2 (A305-502A, rabbit, Thermo Fisher Scientific) and GAPDH (sc-47724, mouse, Santa Cruz) primary antibodies at dilutions of 1:1,000 each. Detection was carried out using ECL (Amersham Biosciences).

Bromodeoxyuridine (BrdU) proliferation assay

HCF proliferation was detected *in situ* using BrdU as described [10]. In brief, HCFs were

labeled with BrdU for 16 hours, and then fixed with ethanol and immunostained for BrdU incorporation using the BrdU-Labeling and Detection kit II (Sigma-Aldrich) according to manufacturer's recommendations. The total number of nuclei was determined by manual counting of DAPI-stained nuclei in 6 random fields per coverslip (original magnification, ×20). All BrdU-positive nuclei were counted in each coverslip. Digital photographs of fluorescence were acquired with a Keyence microscope (BZ-X810) and processed with Adobe Photoshop.

Wound migration assay

HCF migration was detected as previously described [10]. In brief, $1X10^4$ HCFs were plated onto each well of a 2-well Culture-Insert 35 mm μ-Dish (81176, Ibidi, Fitchburg, WI). Once the cells were confluent and high-cell density/confluency led to contact inhibition of proliferation, the silicone insert on each dish was removed to reveal a defined cell-free gap. The medium was replaced, and images were taken at 0 and 24 hours. Subsequently, the distance between cell fronts was quantified in three wells of each group using ImageJ software. Initial open (cell-free) areas (0 hour) were measured to serve as the total open area, and the percentage of open area after 24 hours was calculated to determine the migratory potential of HCFs.

Apoptosis by TUNEL staining

DNA fragmentation was detected *in situ* using TUNEL [16]. In brief, CM were incubated with proteinase K, and DNA fragments were labeled with fluorescein-conjugated dUTP using terminal deoxynucleotidyl transferase (Roche Diagnostics). The total number of nuclei was determined by manual counting of DAPI-stained blue nuclei in 6 random fields per slide or coverslip (original magnification, ×200). All TUNEL-positive green nuclei were then counted. Digital photographs of fluorescence were acquired with a Keyence microscope (BZ-X810) and processed with Adobe Photoshop.

Caspase-Glo 3/7 assay

Caspase 3/7 activity in CMs was measured with the Caspase-Glo 3/7 Assay kit (G8090, Promega) for detecting apoptosis. Briefly, 10 µg of protein in a 50 µl total volume was mixed with 50 µl of caspase-Glo 3/7 reagent and incubated for 1 hour at room temperature. Luminescence was measured with the Synergy LX FA Multi-Mode Microplate Reader (BioTek Instruments), with each CM sample run in triplicate as described [1, 5].

***In silico* analyses to identify conserved transcription factor binding sites in MIAT promoters and electrophoretic mobility shift assays (EMSAs)**

To identify conserved DNA regions in MIAT promoters, we performed homology searches between the 10-kb MIAT human and mouse promoter regions. We used the TFSearch, TRANSFAC, ConTra, and MacVector subsequence search programs to identify potential transcription factor binding sites in the MIAT conserved regions.

Nuclear protein extracts were prepared after transfection of CMV-BACH2 in AC16 cells as described previously [13]. EMSAs were then performed with a kit (E33075, Thermo Fisher Scientific). In brief, labeled double-stranded oligonucleotides and 5µg of nuclear extracts that were prepared from transfected AC16 cells were incubated according to the manufacturer's instructions. We used the following double-stranded oligonucleotides including two conserved BACH2 binding regions in human and mouse MIAT promoters: E1 forward primer, 5'-GATCCCACAATC***TCTGAGTCAGCAGC***CACCAGG-3', E1 reverse primer, 5'-GATCCCTGGTG***GCTGCTGACTCAGA***GATTGTGG-3', E2 forward primer, 5'-GATCCTTCCTC***TTCTCTGACTCAAGT***CACTGAA-3', and E2 reverse primer, 5'-GATCTTCAGTG***ACTTGAGTCAGAGAA***GAGGAAG-3'. The BACH2 protein-DNA complex was separated from unbound labeled DNA using nondenaturing polyacrylamide electrophoresis, and this protein-DNA complex was visualized as described in the EMSA kit. For antibody reactions, we preincubated nuclear extracts with 1µl of rabbit polyclonal BACH2 antibody (A305-502A,

Thermo Fisher Scientific) for 30 minutes at room temperature prior to adding the nuclear extract to the binding reaction as described previously [11, 13]. For antibody specificity control, the nuclear extract was preincubated with 1µl of rabbit IgG antibody.

Supplementary References

1. Bayoumi AS, Teoh JP, Aonuma T, Yuan Z, Ruan X, Tang Y, et al. MicroRNA-532 protects the heart in acute myocardial infarction, and represses prss23, a positive regulator of endothelial-to-mesenchymal transition. Cardiovasc Res. 2017;113(13):1603-14. doi: 10.1093/cvr/cvx132.

2. Bayoumi AS, Park KM, Wang Y, Teoh JP, Aonuma T, Tang Y, et al. A carvedilol-responsive microRNA, miR-125b-5p protects the heart from acute myocardial infarction by repressing pro-apoptotic bak1 and klf13 in cardiomyocytes. J Mol Cell Cardiol. 2017;114:72-82. doi: 10.1016/j.yjmcc.2017.11.003.

3. Aonuma T, Moukette B, Kawaguchi S, Barupala NP, Sepulveda MN, Frick K, et al. MiR-150 attenuates maladaptive cardiac remodeling mediated by long noncoding RNA MIAT and directly represses profibrotic Hoxa4. Circ Heart Fail. 2022;15(4):e008686. doi: 10.1161/CIRCHEARTFAILURE.121.008686.

4. Kawaguchi S, Moukette B, Sepulveda MN, Hayasaka T, Aonuma T, Haskell AK, et al. SPRR1A is a key downstream effector of MiR-150 during both maladaptive cardiac remodeling in mice and human cardiac fibroblast activation. Cell Death Dis. 2023;14(7):446. doi: 10.1038/s41419-023-05982-y.

5. Tang Y, Wang Y, Park KM, Hu Q, Teoh JP, Broskova Z, et al. MicroRNA-150 protects the mouse heart from ischaemic injury by regulating cell death. Cardiovasc Res. 2015;106(3):387-97. doi: 10.1093/cvr/cvv121.

6. Ramakrishna S, Kim IM, Petrovic V, Malin D, Wang IC, Kalin TV, et al. Myocardium defects and ventricular hypoplasia in mice homozygous null for the Forkhead Box M1 transcription factor. Developmental dynamics : an official publication of the American Association of Anatomists. 2007;236(4):1000-13. doi: 10.1002/dvdy.21113.

7. Park KM, Teoh JP, Wang Y, Broskova Z, Bayoumi AS, Tang Y, et al. Carvedilol-responsive microRNAs, miR-199a-3p and -214 protect cardiomyocytes from simulated ischemia-reperfusion injury. Am J Physiol Heart Circ Physiol. 2016;311(2):H371-83. doi: 10.1152/ajpheart.00807.2015.

8. Kim IM, Tilley DG, Chen J, Salazar NC, Whalen EJ, Violin JD, et al. Beta-blockers alprenolol and carvedilol stimulate beta-arrestin-mediated EGFR transactivation. Proc Natl Acad Sci U S A. 2008;105(38):14555-60. doi: 10.1073/pnas.0804745105.

9. Aurora AB, Mahmoud AI, Luo X, Johnson BA, van Rooij E, Matsuzaki S, et al. MicroRNA-214 protects the mouse heart from ischemic injury by controlling Ca(2)(+) overload and cell death. The Journal of clinical investigation. 2012;122(4):1222-32. doi: 10.1172/JCI59327.

10. Teoh JP, Bayoumi AS, Aonuma T, Xu Y, Johnson JA, Su H, et al. Beta-arrestin-biased agonism of beta-adrenergic receptor regulates Dicer-mediated microRNA maturation to promote cardioprotective signaling. J Mol Cell Cardiol. 2018;118:225-36. doi: 10.1016/j.yjmcc.2018.04.001.

11. Kim IM, Ramakrishna S, Gusarova GA, Yoder HM, Costa RH, Kalinichenko VV. The forkhead box m1 transcription factor is essential for embryonic development of pulmonary vasculature. J Biol Chem. 2005;280(23):22278-86. doi: 10.1074/jbc.M500936200.

12. Kim IM, Wolf MJ, Rockman HA. Gene deletion screen for cardiomyopathy in adult Drosophila identifies a new notch ligand. Circulation research. 2010;106(7):1233-43. doi: 10.1161/CIRCRESAHA.109.213785.

13. Kim IM, Zhou Y, Ramakrishna S, Hughes DE, Solway J, Costa RH, et al. Functional characterization of evolutionarily conserved DNA regions in forkhead box f1 gene locus. J Biol Chem 2005;280(45):37908-16. doi: 10.1074/jbc.M506531200.

14. Noma T, Lemaire A, Naga Prasad SV, Barki-Harrington L, Tilley DG, Chen J, et al. Beta-arrestin-mediated beta1-adrenergic receptor transactivation of the EGFR confers cardioprotection. J Clin Invest. 2007;117(9):2445-58. doi: 10.1172/JCI31901.

15. Tilley DG, Kim IM, Patel PA, Violin JD, Rockman HA. beta-Arrestin mediates beta1-adrenergic receptor-epidermal growth factor receptor interaction and downstream signaling. J Biol Chem 2009;284(30):20375-86. doi: 10.1074/jbc.M109.005793.

16. Rakesh K, Yoo B, Kim IM, Salazar N, Kim KS, Rockman HA. beta-Arrestin-biased agonism of the angiotensin receptor induced by mechanical stress. Science signaling. 2010;3(125):ra46. doi: 10.1126/scisignal.2000769.

Table S1. BACH2 binding sites in human and mouse MIAT promotors

Human

Matrix ID	Name	Score	Relative score	Sequence ID	Start	End	Strand	Predicted sequence
MA1101.1	BACH2	10.6496	0.841592675871	MIAT	-7505	-7492	+	GGTGATACAGCACA
MA1101.1	BACH2	8.97628	0.813475987974	MIAT	-6856	-6843	-	CCTGCCTCAGCCTC
MA1101.1	BACH2	10.5599	0.840084672089	MIAT	-5502	-5489	-	TATGAATCATCAGG
MA1101.1	BACH2	9.24844	0.818048985247	MIAT	-5713	-5700	-	TATGATCCAGCAAT
MA1101.1	BACH2	8.97628	0.813475987974	MIAT	-6061	-6048	+	CCTGCCTCAGCCTC
MA1101.1	BACH2	8.97628	0.813475987974	MIAT	-2578	-2565	+	CCTGCCTCAGCCTC
MA1101.2	BACH2	4.8388	0.811968899206	MIAT	-6903	-6885	-	TTGCAGTGACTTATGATTG
MA1101.2	BACH2	4.47638	0.808608969111	MIAT	-6903	-6885	+	CAATCATAAGTCACTGCAA
MA1101.2	BACH2	4.32642	0.807218710831	MIAT	-5503	-5485	-	GCATTATGAATCATCAGGA
MA1101.2	BACH2	3.84528	0.802758182807	MIAT	-5062	-5044	-	CAGGCATGAGCCACCGCGC
MA1101.1	BACH2	15.2674	0.919183194493	MIAT	-3391	-3378	+	TCTGAGTCAGCAGC
MA1101.1	BACH2	12.2537	0.868545463285	MIAT	-3394	-3381	-	GCTGACTCAGAGAT
MA1101.1	BACH2	8.97628	0.813475987974	MIAT	-8038	-8025	+	CCTGCCTCAGCCTC
MA1101.2	BACH2	4.2192	0.806224646763	MIAT	-2412	-2394	+	TAGGCGTGAGCCACCATGC
MA1101.2	BACH2	4.10951	0.805207811795	MIAT	-1508	-1490	-	CTCCTGTGGGTCATCCTTC
MA1101.2	BACH2	-	-	MIAT	-3016	-3005	+	TTCTCTGACTCAAGT

Mouse

Matrix ID	Name	Score	Relative score	Sequence ID	Start	End	Strand	Predicted sequence
MA1101.1	BACH2	10.8516	0.84498541197	MIAT	-9882	-9869	-	AGTGACCCAGCAGG
MA1101.1	BACH2	9.70367	0.825698032698	MIAT	-7429	-7416	-	TCTGCCTCAGCTCT
MA1101.1	BACH2	9.21719	0.817524016999	MIAT	-8098	-8085	-	GCTCACTCAGCCTC
MA1101.1	BACH2	9.15582	0.816492732651	MIAT	-8563	-8550	-	ACTGAATCACCAAA
MA1101.1	BACH2	8.95331	0.813090061558	MIAT	-7302	-7289	-	AGTTACACAGCAGC
MA1101.1	BACH2	8.87405	0.811758387882	MIAT	-8751	-8738	+	**ACTGAGCCAGCAGG**
MA1101.2	BACH2	4.61461	0.809890505295	MIAT	-7899	-7881	-	CACCCAAGACTCACCCACT
MA1101.1	BACH2	18.0032	0.965151623089	MIAT	-5848	-5835	-	CCTGACTCAGCAAC
MA1101.1	BACH2	10.8619	0.845159130135	MIAT	-4881	-4868	+	**AATGAGTCAGTAGG**
MA1101.1	BACH2	9.20876	0.817382315152	MIAT	-4884	-4871	-	ACTGACTCATTAAG
MA1101.1	BACH2	9.15727	0.816517089409	MIAT	-5845	-5832	+	**GCTGAGTCAGGGGC**
MA1101.2	BACH2	5.16596	0.815001911496	MIAT	-6641	-6623	-	AGACAATGCCTCACAGCAG
MA1101.2	BACH2	4.4971	0.808801034205	MIAT	-6641	-6623	+	CTGCTGTGAGGCATTGTCT
MA1101.2	BACH2	9.39532	0.854211567041	MIAT	-4003	-3985	-	TCAGCATGAGTCAGATCAG
MA1101.1	BACH2	9.90215	0.829033001618	MIAT	-4002	-3989	-	CATGAGTCAGATCA
MA1101.2	BACH2	5.91122	0.821911087119	MIAT	-4003	-3985	+	CTGATCTGACTCATGCTGA
MA1101.1	BACH2	9.14348	0.816285459848	MIAT	-1672	-1659	-	GATTGCTCAGCACT
MA1101.2	BACH2	4.99477	0.813414902206	MIAT	-2971	-2953	-	CAAATGGGAGTCATCGTGA
MA1101.2	BACH2	-	-	MIAT	-4068	-4057	+	GTCAGTGACTCAAACAG

Table S2. Demographic characteristics of human left ventricle samples

Etiology	Non-failing	HFrEF
Number	6	6
Age (years)	75.17 ± 5.70	55.50 ± 4.75
LVEF (%)	64 ± 0.02	24 ± 3.00***

****P* <0.001 vs. non-failing. HFrEF: Heart failure with reduced ejection fraction. LVEF: Left ventricular ejection fraction.

Figure S1

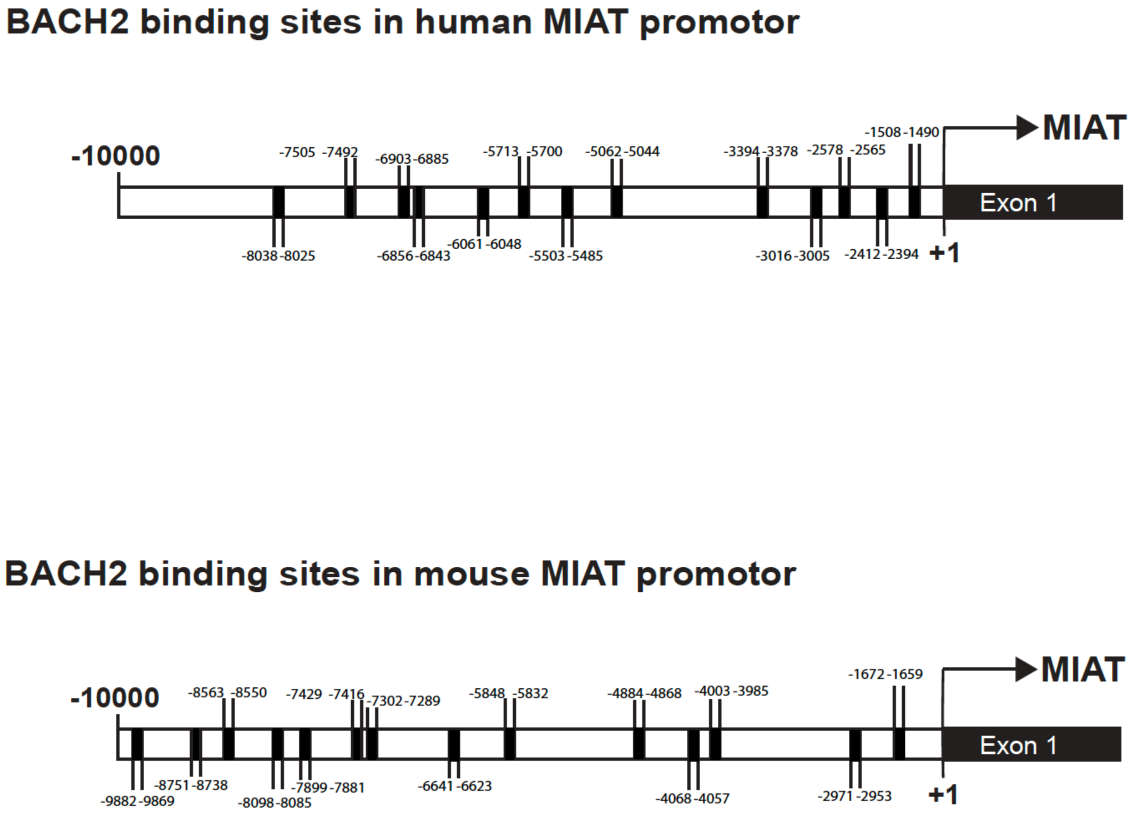

Figure S1. Potential BACH2-binding sites in human and mouse *MIAT* promoters. Potential BACH2 binding sites are shown as black boxes and determined by MacVector Subsequence searches, TFSearch: www.cbrc.jp/research/db/TFSEARCH.html, and TRANSFAC (String-based search query): www.cbi-l.upenn.edu/tess/. The detailed locations of BACH2 binding sites are listed in **Table S1**.

Figure S2

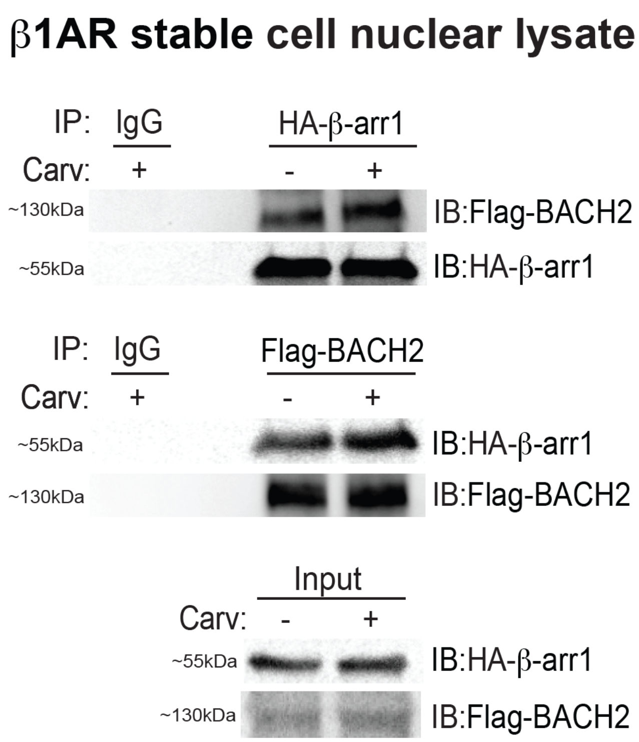

Figure S2. Carvedilol induces the nuclear interaction of β-arrestin1 with BACH2. β_1-adrenergic receptor (β_1AR) stable cells were transfected with HA-β-arrestin1 (β-arr1) and Flag-BACH2 constructs. After carvedilol (Carv) treatment for 16 hours, nuclear extracts (NEs) were prepared and subjected to immunoprecipitation (IP) with anti-Flag, anti-HA, or nonspecific IgG (control). Interaction of BACH2 with β-arr1 was examined by immunoblotting (IB) with anti-HA and anti-Flag. NEs were also immunoblotted with HA and Flag antibodies for input.

Figure S3

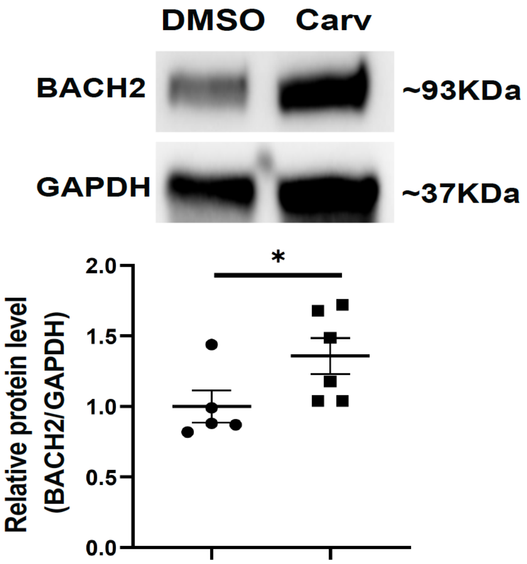

Figure S3. **Carvedilol increases the cardiac levels of BACH2.** BACH2 protein levels were measured in left ventricles from wild-type mice treated with carvedilol (Carv: 19 mg/kg per day) or vehicle for 7 days. N=5–6 per group. Data are shown as the fold induction of BACH2 levels normalized to GAPDH. Unpaired 2-tailed t-test. *P<0.05 vs. DMSO.

Figure S4

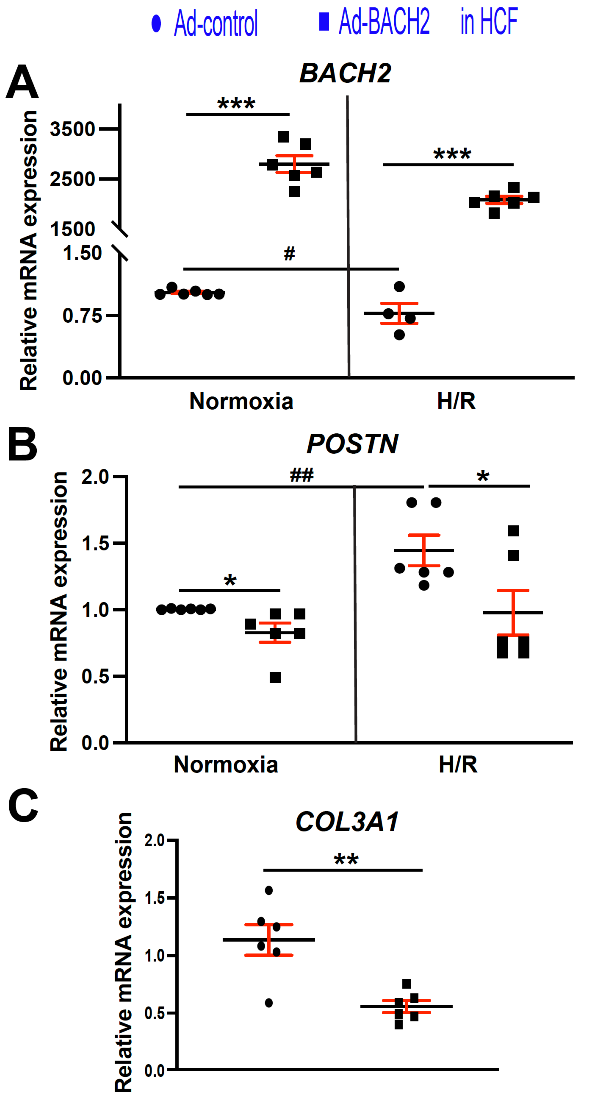

Figure S4. *BACH2* is downregulated in primary adult human cardiac fibroblasts after hypoxia/reoxygenation and decreases the expression of fibrotic markers in HCFs. A–C, Primary adult human cardiac fibroblasts (HCFs) were infected with control adenovirus (Ad-control) or adenovirus expressing human BACH2 (Ad-BACH2). Infected HCFs were then subjected to hypoxia/reoxygenation (H/R). Real-Time Quantitative Reverse Transcription (QRT)-PCR for *BACH2* (**A**), *POSTN* (**B**), or *COL3A1* (**C**) were performed to check their expression after the indicated infection. Data were normalized to *GAPDH* and are expressed relative to Ad-control. N=4–6 per group. Two-way ANOVA with Tukey's multiple comparison test (**A–B**) or Unpaired 2-tailed t-test (**C**). $^{*}P<0.05$, $^{**}P<0.01$, or $^{***}P<0.001$ vs. Ad-control. $^{\#}P<0.05$ or $^{\#\#}P<0.01$ vs. normoxia. Data are presented as the mean ± SEM.

Figure S5

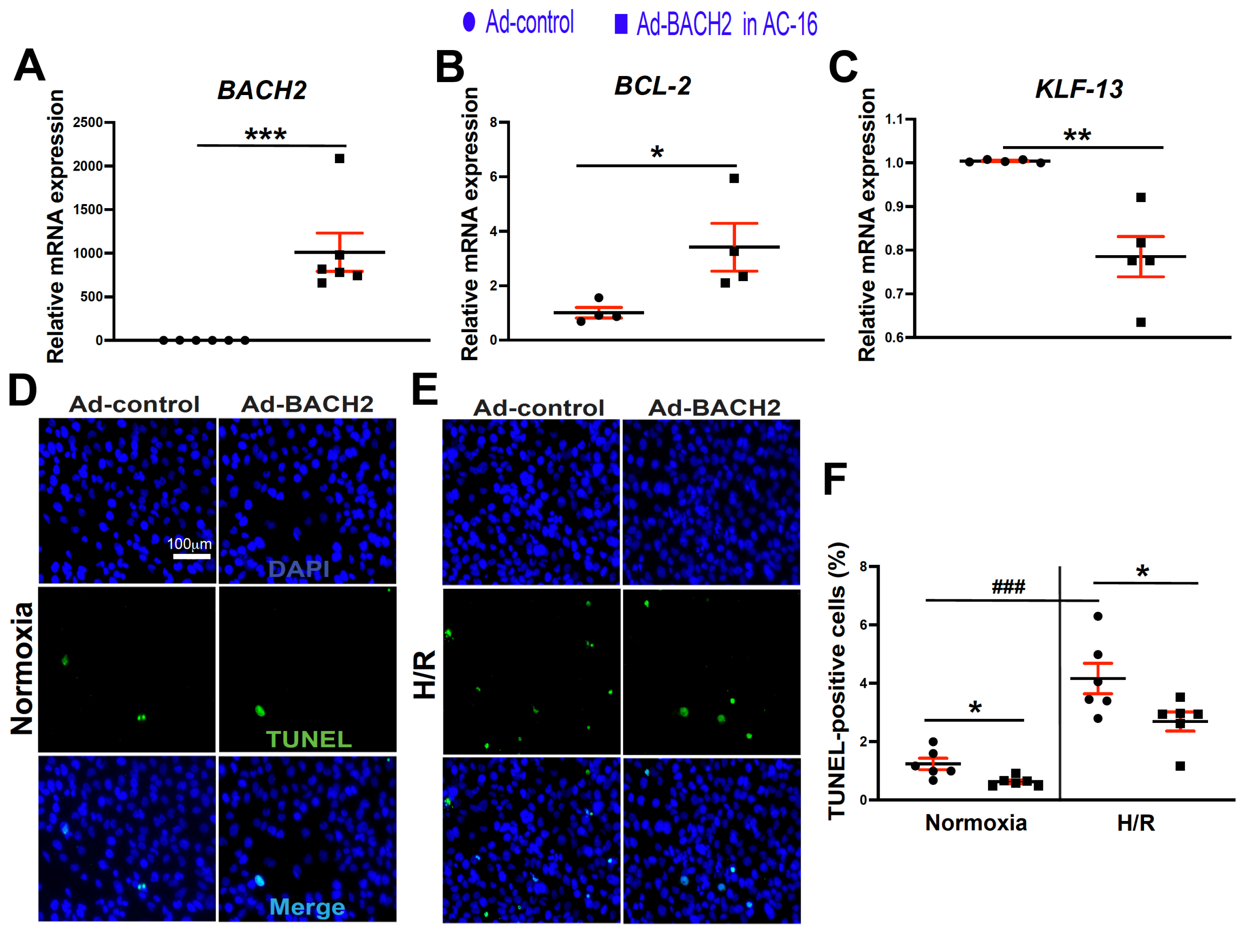

Figure S5*. BACH2* overexpression decreases human cardiomyocyte apoptosis. A–C, AC16 cells were infected with control adenovirus (Ad-control) or adenovirus expressing human BACH2 (Ad-BACH2). QRT-PCR for *BACH2* (**A**), anti-apoptotic *BCL-2* (**B**), or pro-apoptotic *KLF-13* (**C**) were then performed to check their expression after the indicated infection. Data were normalized to *GAPDH* and are expressed relative to Ad-control. N=4–6 per group. Unpaired 2-tailed t-test. **P*<0.05, ***P*<0.01, or ****P*<0.001 vs. Ad-control. **D–F,** AC16 cells, which were infected with Ad-control or Ad-BACH2, were subjected to H/R. TUNEL assays were also conducted in both normoxic and H/R conditions. Scale bar = 100 µm. The percentage of apoptotic nuclei (green) was calculated after the normalization of total nuclei (blue). N = 6 per group. Two-way ANOVA with Tukey's multiple comparison test. **P*<0.05 vs. Ad-control. ###*P*<0.001 vs. normoxia. Data are presented as the mean ± SEM.

Figure S6

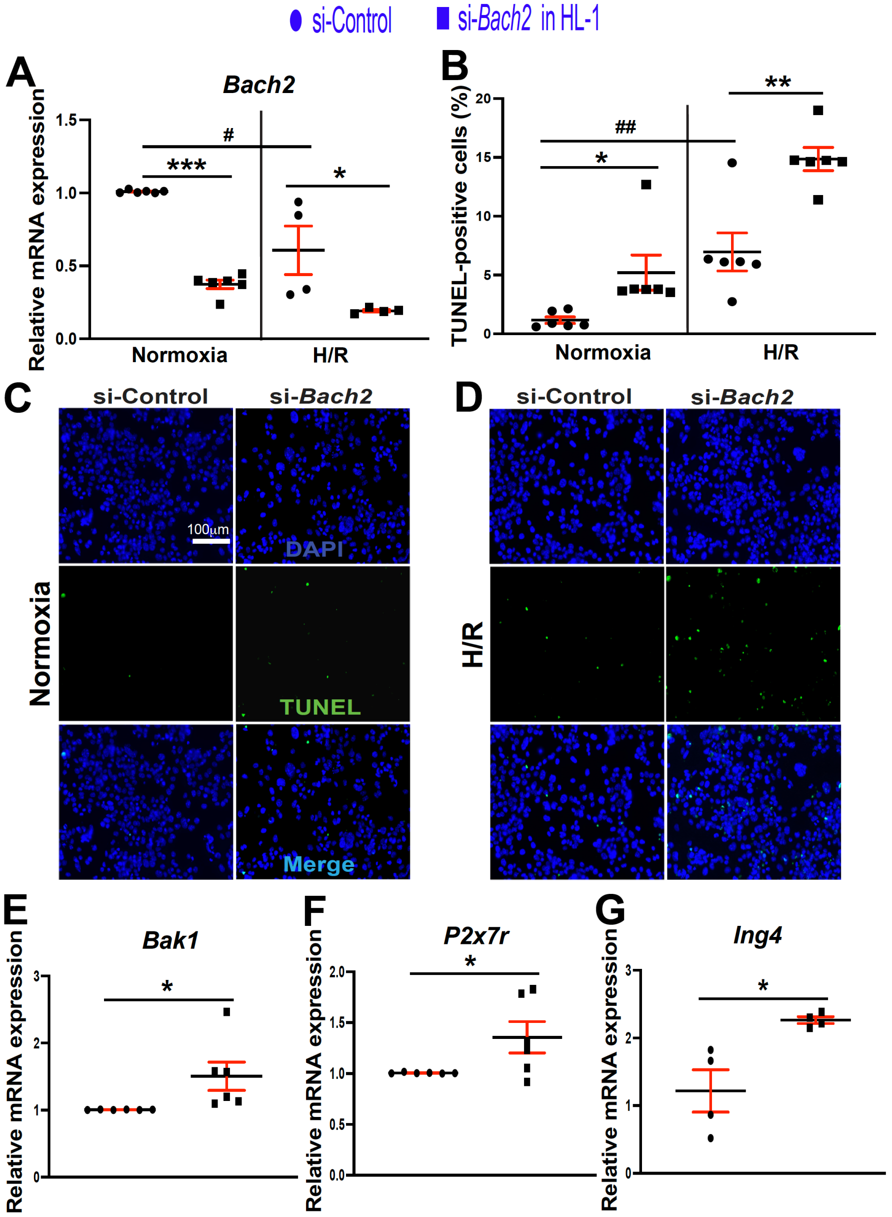

Figure S6*. Bach2* is decreased in ischemic mouse cardiomyocytes, and *Bach2* knockdown increases apoptosis in mouse cardiomyocytes. A, HL-1 cells, which were transfected with si-Control or si-*Bach2*, were subjected to H/R. QRT-PCR for *Bach2* was conducted to check the knockdown efficiency. Data were normalized to *Gapdh* and expressed relative to si-Controls. N = 4–6 per group. Two-way ANOVA with Tukey's multiple comparison test. **P*<0.05 or ****P*<0.001 vs. si-Control. #*P*<0.05 vs. normoxia. **B–D,** TUNEL assays were also conducted in transfected HL-1 cells after normoxic and H/R conditions. Scale bar = 100 μm. The percentage of apoptotic nuclei (green) was calculated after the normalization of total nuclei (blue). N = 6 per group. Two-way ANOVA with Tukey's multiple comparison test. **P*<0.05 or ***P*<0.01 vs. si-Control. ##*P*<0.01 vs. normoxia. **E–G,** HL-1 cells were transfected with si-Control or si-*Bach2* and processed for QRT-PCR expression analyses of pro-apoptotic *Bak1, P2x7r,* and *Ing4*. N = 4–6 per group. Unpaired two-tailed t-test. **P*<0.05 vs. si-Control. All data are shown as mean ± SEM.
