# Supplementary material for: BACH2 links β1-adrenergic receptor/β-arrestin1 signaling to MIAT to inhibit cardiac fibroblast activation and cardiomyocyte apoptosis
Source: Cell Death Discov. 2026 Feb 28;12:127. doi: 10.1038/s41420-026-02985-4 (PMC13039337; doi:10.1038/s41420-026-02985-4)

Complete unedited gels for Figure 2C

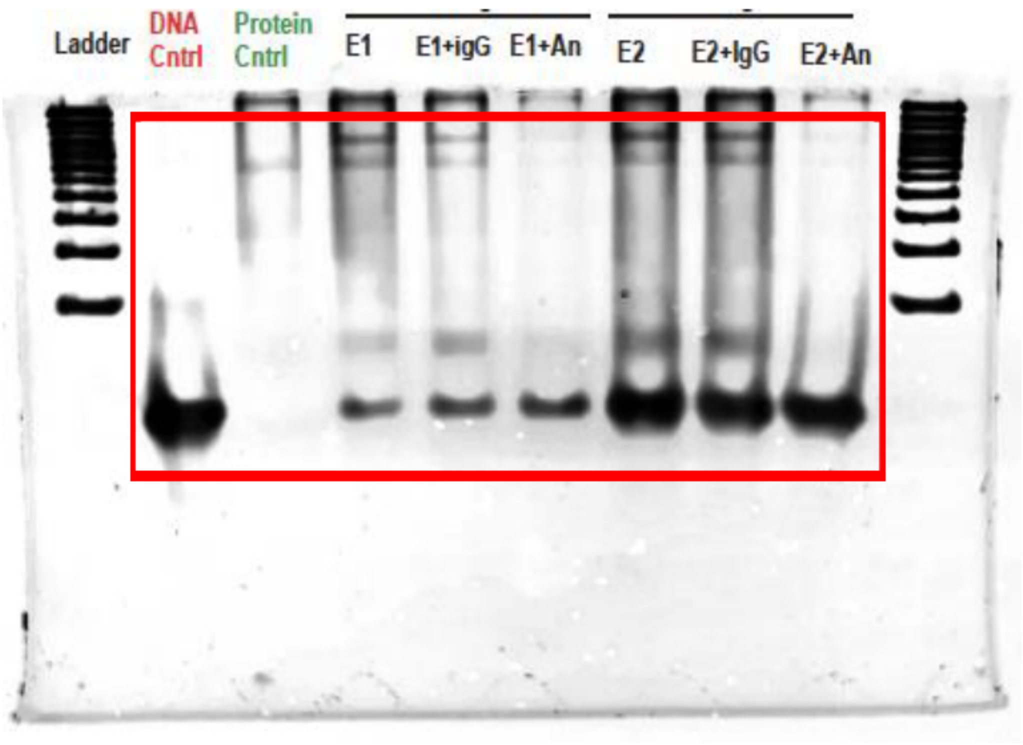

Complete unedited blots for Figure S2

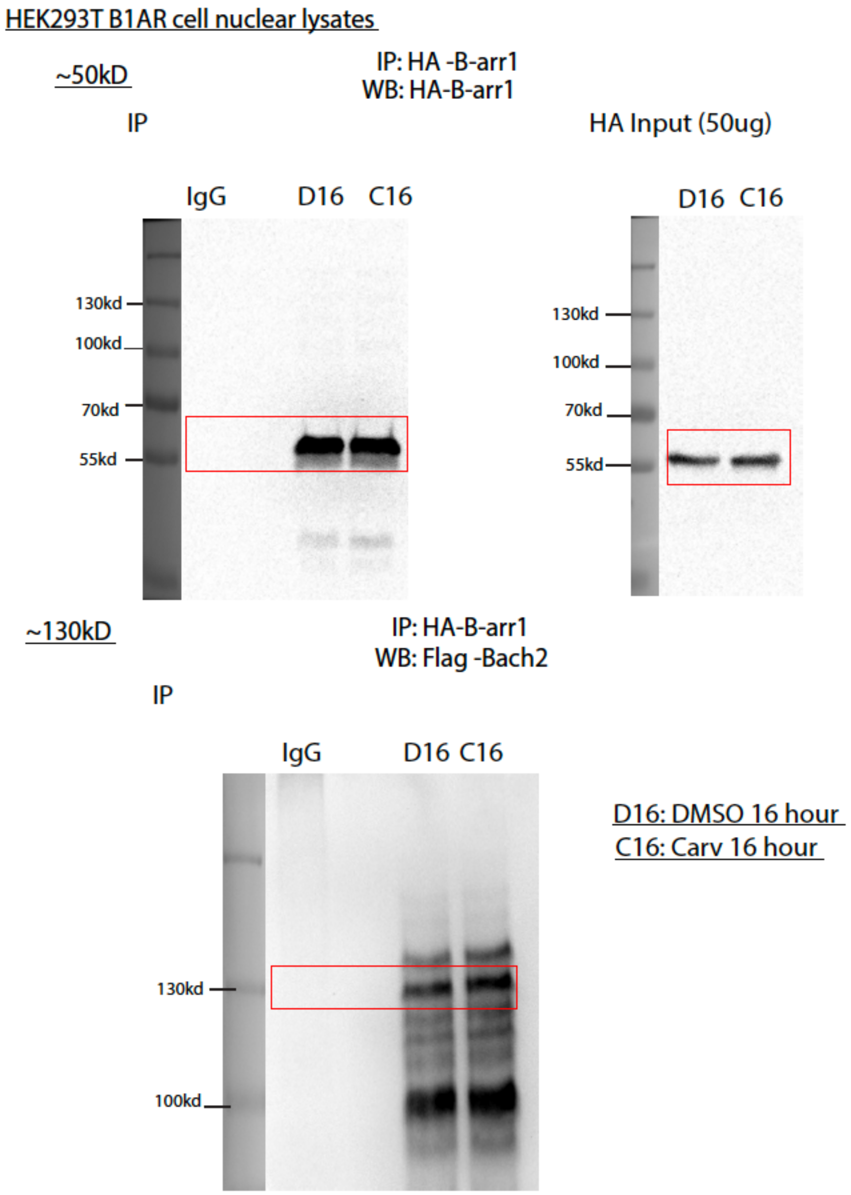

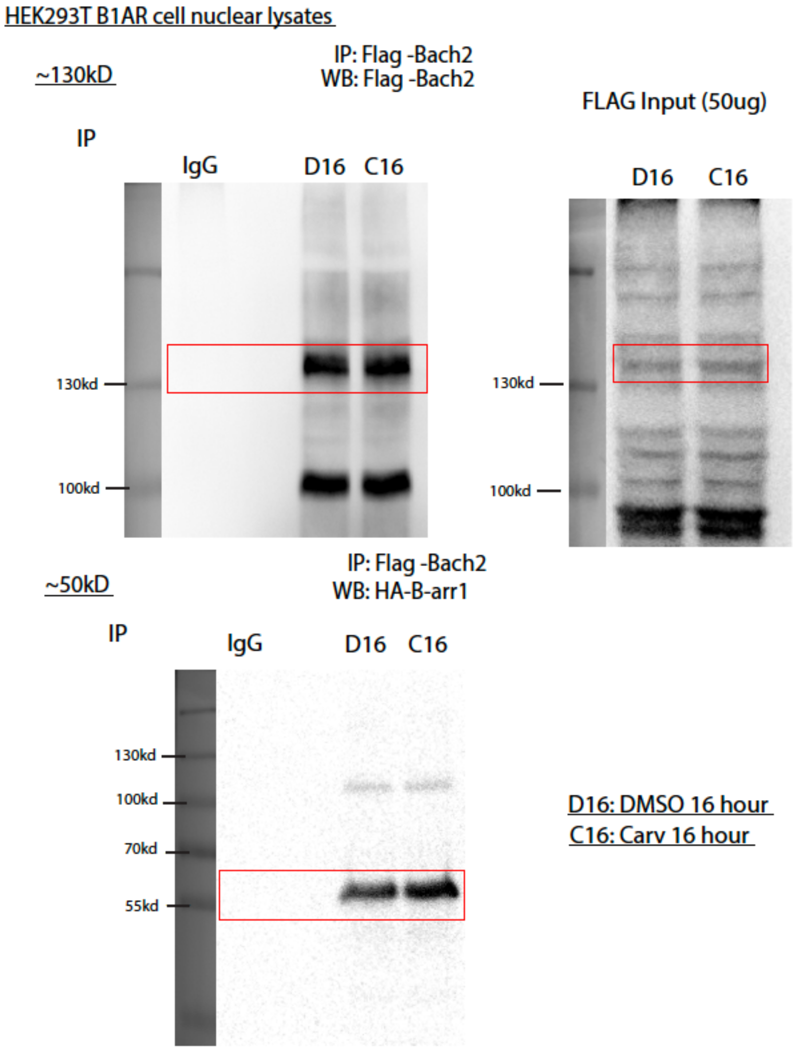
HEK293T B1AR cell nuclear lysates
~130kD
IP: Flag -Bach2
WB: Flag -Bach2
IP
IgG
D16
C16
130kd
100kd
FLAG Input (50ug)
D16
C16
130kd
100kd
~50kD
IP: Flag -Bach2
WB: HA-B-arr1
IP
IgG
D16
C16
130kd
100kd
70kd
55kd
D16: DMSO 16 hour
C16: Carv 16 hour

Complete unedited blots for Figure S3

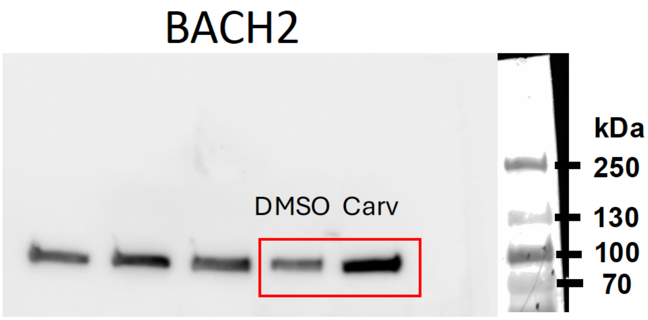

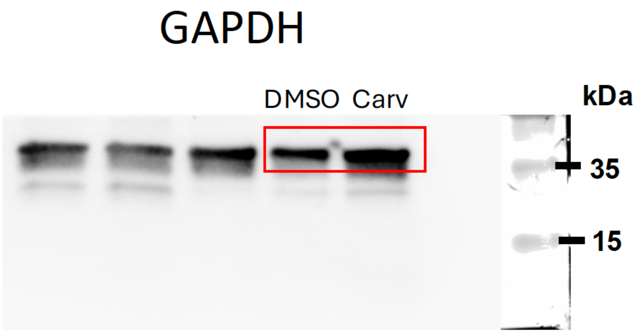

Supplement: Supplementary file 2 — Unedited Gels and Blots [file 41420_2026_2985_MOESM2_ESM.pdf]
